# Reproducibility and accuracy of microscale thermophoresis in the NanoTemper Monolith: a multi laboratory benchmark study

**DOI:** 10.1007/s00249-021-01532-6

**Published:** 2021-04-21

**Authors:** Blanca López-Méndez, Bruno Baron, Chad A. Brautigam, Thomas A. Jowitt, Stefan H. Knauer, Stephan Uebel, Mark A. Williams, Arthur Sedivy, Olga Abian, Celeste Abreu, Malgorzata Adamczyk, Wojciech Bal, Sylvie Berger, Alexander K. Buell, Carlo Carolis, Tina Daviter, Alexander Fish, Maria Garcia-Alai, Christian Guenther, Josef Hamacek, Jitka Holková, Josef Houser, Chris Johnson, Sharon Kelly, Andrew Leech, Caroline Mas, Daumantas Matulis, Stephen H. McLaughlin, Roland Montserret, Rouba Nasreddine, Reine Nehmé, Quyen Nguyen, David Ortega-Alarcón, Kathryn Perez, Katja Pirc, Grzegorz Piszczek, Marjetka Podobnik, Natalia Rodrigo, Jasmina Rokov-Plavec, Susanne Schaefer, Tim Sharpe, June Southall, David Staunton, Pedro Tavares, Ondrej Vanek, Michael Weyand, Di Wu

**Affiliations:** 1 Departamento de Bioquímica y Biología Molecular y Celular-Institute of Biocomputation and Physics of Complex Systems (BIFI), Instituto Aragonés de Ciencias de la Salud (IACS), Instituto de Investigación Sanitaria Aragón (IIS Aragón), Universidad de Zaragoza, C/ Mariano Esquillor S/N, 50018 Zaragoza, Spain; 2 Department of Biochemistry, Faculty of Science, Charles University, Hlavova 8, 128 43 Prague, Czech Republic; 3 Faculty of Chemistry, Chair of Drug and Cosmetics Biotechnology, Warsaw University of Technology, ul. Noakowskiego 3, 00-664 Warsaw, Poland; 4 Institute of Biochemistry and Biophysics, PAS, Pawinskiego 5a, 02-106 Warsaw, Poland; 5 Molecular Biophysics, Institut Pasteur, 25-28 Rue du Dr Roux, 75015 Paris, France; 6 Institut de Recherches Servier, 125, Chemin de Ronde, 78290 Croissy-sur-Seine, France; 7 Departments of Biophysics and Microbiology, UT Southwestern Medical Center, Dallas, TX 75390 USA; 8 Department of Biotechnology and Biomedicine, Technical University of Denmark, Søltofts Plads, Kgs., 2800 Lyngby, Denmark; 9 BioMolecular Screening and Protein Technologies Unit, Centre for Genomic Regulation (CRG), Dr. Aiguader St, 88, 08003 Barcelona, Spain; 10 Department of Biological Sciences, BiophysX Centre, Institute of Structural and Molecular Biology, Birkbeck, University of London, Malet Street, London, WC1E 7HX UK; 11 Present Address: Shared Research Facilities, The Institute of Cancer Research, London, SW7 3RP UK; 12 Department of Biochemistry, Netherlands Cancer Institute, Plesmanlaan 121, 1066CX Amsterdam, Netherlands; 13 EMBL-Hamburg, Notkestrasse 85, 22607 Hamburg, Germany; 14 Center for Molecular Biophysics, UPR 4301 CNRS Orléans, Rue Charles Sadron, 45071 Orléans, France; 15 Glycobiochemistry and Biomolecular Interaction and Crystallization Core Facility, CEITEC MU, Kamenice 5, 625 00 Brno, Czech Republic; 16 MRC Laboratory of Molecular Biology, Francis Crick Avenue, Cambridge Biomedical Campus, Cambridge, CB2 0QH UK; 17 Biomolecular Analysis Core Facility, University of Manchester, Oxford Rd, Manchester, M13 9PL UK; 18 Institute of Molecular, Cell and Systems Biology, University of Glasgow, B4-13 Joseph Black Building, G12 8QQ Glasgow, Scotland, UK; 19 Biochemistry IV-Biopolymers, University of Bayreuth, Universitaetsstr. 30, 95447 Bayreuth, Germany; 20 Department of Biology, Bioscience Technology Facility, University of York, Wentworth Way, York, YO10 5DD UK; 21 Biophysics Platform, Novo Nordisk Foundation Center for Protein Research, University of Copenhagen, 2200 Copenhagen, Denmark; 22 Integrated Structural Biology Grenoble (ISBG), UMS 3518 (CNRS-CEA-UGA-EMBL), 71 avenue des Martyrs, 38042 Grenoble Cedex 9, France; 23 Department of Biothermodynamics and Drug Design, Life Sciences Center, Institute of Biotechnology, Vilnius University, Sauletekio StSaulėtekio 7, 10257 Vilnius, Lithuania; 24 Institut de Biologie et Chimie des protéines, CNRS, Université de Lyon, 7 passage du Vercors, 69367 cedex 07 Lyon, France; 25 Institut de Chimie Organique et Analytique (ICOA), CNRS FR 2708, UMR 7311, Université d’Orléans, Orléans, France; 26 Institute of Biocomputation and Physics of Complex Systems (BIFI), Universidad de Zaragoza, C/ Mariano Esquillor S/N, 50018 Zaragoza, Spain; 27 Biophysics Lab, Protein Expression and Purification Core Facility, EMBL Heidelberg, Meyerhofstraße 1, 69117 Heidelberg, Germany; 28 Department of Molecular Biology and Nanobiotechnology, National Institute of Chemistry, Hajdrihova 19, 1000 Ljubljana, Slovenia; 29 NHLBI Biophysics Core Facility, NHLBI/NIH, 50 South Dr, Bethesda, MD 20892 USA; 30 Division of Biochemistry, Department of Chemistry, Faculty of Science, University of Zagreb, Horvatovac 102a, 10000 Zagreb, Croatia; 31 Department of Biochemistry, University of Bayreuth, Universitätsstr. 30, 95447 Bayreuth, Germany; 32 ProteinTechnology, Vienna Biocenter Core Facilities GmbH, Dr. Bohr-Gasse 3, 1030 Vienna, Austria; 33 Biozentrum, University of Basel, Klingelbergstrasse 50/70, 4056 Basel, Switzerland; 34 Department of Biochemistry, University of Oxford, South Parks Rd, Oxford, OX13 5LA UK; 35 Molecular Biophysics Research Laboratory, Departamento de Química, UCIBIO/Requimte, Faculdade de Ciências e Tecnologia, UNL, Campus Caparica, 2829-516 Costa da Caparica, Portugal; 36 Max Planck Institute of Biochemistry, Am Klopferspitz 18, Martinsried, 82152 Planegg, Germany; 37 Department of Biological Sciences, ISMB BiophysX Centre, Institute of Structural and Molecular Biology, Birkbeck, University of London, London, WC1E 7HX UK

**Keywords:** MST, TRIC, Benchmark, Thermophoresis, *K*_D_, Interaction

## Abstract

Microscale thermophoresis (MST), and the closely related Temperature Related Intensity Change (TRIC), are synonyms for a recently developed measurement technique in the field of biophysics to quantify biomolecular interactions, using the (capillary-based) NanoTemper Monolith and (multiwell plate-based) Dianthus instruments. Although this technique has been extensively used within the scientific community due to its low sample consumption, ease of use, and ubiquitous applicability, MST/TRIC has not enjoyed the unambiguous acceptance from biophysicists afforded to other biophysical techniques like isothermal titration calorimetry (ITC) or surface plasmon resonance (SPR). This might be attributed to several facts, e.g., that various (not fully understood) effects are contributing to the signal, that the technique is licensed to only a single instrument developer, NanoTemper Technology, and that its reliability and reproducibility have never been tested independently and systematically. Thus, a working group of ARBRE-MOBIEU has set up a benchmark study on MST/TRIC to assess this technique as a method to characterize biomolecular interactions. Here we present the results of this study involving 32 scientific groups within Europe and two groups from the US, carrying out experiments on 40 Monolith instruments**,** employing a standard operation procedure and centrally prepared samples. A protein–small molecule interaction, a newly developed protein–protein interaction system and a pure dye were used as test systems. We characterized the instrument properties and evaluated instrument performance, reproducibility, the effect of different analysis tools, the influence of the experimenter during data analysis, and thus the overall reliability of this method.

**Supplementary Information:**

The online version contains supplementary material available at 10.1007/s00249-021-01532-6.

## Introduction

The NanoTemper Monolith was introduced as a commercial instrument in 2011, following the accomplishments of academic studies in the years 2006–2010 (Duhr and Braun 2006; Jerabek-Willemsen 2011; Jerabek-Willemsen Jerabek-Willemsen 2014). Despite successive generations of instruments sharing the same name (Monolith NT.115), changes in the hardware, software and best practices in data analysis have occurred over the last 10 years. The current benchmark was designed to characterize the variability of the hardware, the software and data analysis practices independently of each other. To achieve this goal, all sample stocks were centrally prepared. In addition, an exhaustive standard operating procedure (SOP) for the sample preparation and settings for the measurement was prepared, to be followed by each participant of the benchmark (see supplementary material 4).

Possible standards and labeling procedures as well as instrument settings were tested in a small-scale benchmark within the ARBRE-MOBIEU working group prior to the start of this wider benchmark study. To include as many participants as possible and according to our information that most instruments sold contained the red channel, a red dye was chosen and consequently only instruments with red filter sets were eligible.

An invitation with online registration to this benchmark was announced in October 2018 (https://arbre-mobieu.eu/mst-benchmark-call/) within the ARBRE-MOBIEU community.

## Materials and methods

### Buffers

PBS + : PBS (10 mM phosphate buffer pH 7.4, 2.7 mM KCl, 137 mM NaCl) 0.005% Tween-20; for the RED-NHS 2^nd^ generation dye and the lysozyme/nanobody interaction.

Tris + : 20 mM Tris pH 7.8, 150 mM NaCl, 0.005% Tween-20; for the lysozyme/NAG3 interaction.

### Lysozyme labeling procedure

Lysozyme isolated from hen egg white (ROCHE Cat.No. 10837059001) was labeled using the Monolith NT™ Protein Labeling Kit RED-NHS 2^nd^ Generation Amine reactive (NanoTemper Technologies GmbH, MO-L011) following the recommended procedure by the manufacturer.

10 mg of lysozyme were weighed and resuspended in PBS buffer to prepare an initial stock solution at ~ 700 μM.

10 μg of the RED-NHS 2^nd^ generation dye were resuspended and completely dissolved (by briefly vortexing and pipetting up and down) in 25 μl DMSO (Sigma, 34943-M) to obtain a ~ 600 μM solution.

A 100 μL, 20 μM solution of lysozyme was prepared from the initial stock in 1 × labeling buffer (NanoTemper Technologies GmbH) and a 100 μL, 60 μM dye solution (3 × protein concentration) was prepared by mixing 10 μL of the 600 μM dye stock with 90 μl of labeling buffer.

Both lysozyme and dye solutions were mixed in a 1:1 volume ratio (200 μl final volume, 5% DMSO) and incubated for 30 min at room temperature in the dark. Triplicates of this reaction were run in parallel to prepare all red labeled lysozyme samples required for the benchmark.

The gravity flow columns B from the Monolith NT™ Protein Labeling Kit RED-NHS 2^nd^ Generation were equilibrated with the elution, MST/TRIC assay buffer (either PBS + , for the nanobody interaction, or Tris + , for the NAG3 interaction). In brief, the columns were placed with the provided adapters in 15 ml tubes, the top cap of the columns removed, and the storage solution poured off. The columns were then equilibrated and washed three times, with 3 ml MST/TRIC assay buffer each, discarding the flow through. Once the columns were equilibrated with the MST/TRIC assay buffer, 200 μl of the labeling reaction were added to the center of the column and after the sample entered the bed completely, 500 μl of assay buffer were added to the top of the column and the flow through was discarded. The columns were then transferred to new 15 ml collection tubes and the eluates collected after addition of 400 μl of the MST/TRIC assay buffer. The final pooled lysozyme concentration was ~ 3 μM (as measured by its absorbance at 280 nm, corrected by the absorbance of the dye) and the degree of labeling ~ 0.70. One single large stock of lysozyme at 50 nM was prepared by diluting further the labeled lysozyme in the corresponding MST/TRIC assay buffer (PBS + or Tris +) and aliquoted into individual tubes that were labeled and sent to the participants of the benchmark study.

### RED-NHS 2^nd^ generation dye

The RED-NHS 2^nd^ generation dye solution was prepared by diluting the 600 μM dye stock to the final appropriate concentration (25 nM) in PBS + buffer.

### N,N’,N’’-triacetylchitotriose (NAG3)

A NAG3 (Sigma, T2144) initial stock at 10 mM was prepared by weighing and resuspending the NAG3 powder in the MST/TRIC assay Tris + buffer. A single solution at 2 mM was prepared by dilution of this initial stock solution with Tris + buffer. The 2 mM solution was then aliquoted into individual tubes that were labeled and sent to the participants of the benchmark study.

### Nanobody

Several VHH camelid single domain antibodies, often called nanobodies, with different affinities to lysozyme have been produced in the Biomolecular Analysis Core Facility (University of Manchester, Thomas Jowitt) and a detailed description of the selection procedure for these nanobodies can be found within this special issue (Birchenough 2021). Briefly a nanobody was selected for use in this benchmarking study which had an affinity for lysozyme that can be easily detected using both the Pico and Monolith NT115 instruments and by isothermal titration calorimetry. The nanobody is a purely monomeric 14.2 kDa molecule, which was created by mutation of the CDR3 loop of Cab-Lys3 (De Genst et al. 2002) T101 to a serine residue which decreases the affinity of the WT protein from 5 to 112 nM (as measured by ITC). Protein production: The nanobody VHH sequence was engineered into pET-22B expression vector with a C-terminal 6-His tag. The vector was transformed into competent T7-Express *E. coli* cells (New England Biolabs) and selected on 100 µg/ml ampicillin plates. One colony was selected for overnight growth in 5 ml LB broth supplemented with 100 µg/ml ampicillin shaking at 37 °C. The cells were pelleted by centrifugation at 500 rcf for 5 min and resuspended in 5 ml of sterile LB. This suspension was used to inoculate 1 L of Magic Media™ (Thermo Fisher) divided between two 2-L baffled flasks and cells were incubated for 24 h at 28 °C on a rotary shaker set to 180 rpm. Cells were extracted by centrifugation at 9000 rcf for 20 min at 4 °C with 250 ml cell suspension per 500 ml centrifuge tube and resuspended in 50 ml 50 mM phosphate buffered saline with 1% glycerol pH 7.0. Cells were then frozen at − 80 °C until needed. Protein extraction: cells were thawed quickly then left on ice for 20-min before sonication in a Soniprep 150 tissue homogenizer (4 × 15 s) kept on ice. Cell debris was centrifuged at 21,000 rcf at 4 °C for 10 min and the supernatant collected. The supernatant was diluted 1:2 in 10 mM PBS pH 7.4 and injected onto a 5 ml Profinity IMAC column (BioRad) at 4 ml/min using a BioRad NGC FPLC. Protein was eluted in PBS supplemented with 0.5 M ultrapure imidazole (Sigma) without a gradient and collected in deep-well 96-well plates. The elution peak was collected and further purified on a 24/300 Superdex-75 column in PBS plus 0.005% P20 with a flowrate of 0.75 ml/min. Purified nanobody was collected and diluted to 0.25 mg/ml (19 µM) ready for shipping.

### Benchmark logistics

Sample envelopes containing copies of pure dye, NAG3, anti-lysozyme nanobody as well as red labeled lysozyme (both in PBS + and Tris + buffer) (see the protocol/SOP) were centrally prepared and shipped at room temperature to the respective participants together with a pack of premium coated capillaries (MO-K025) and a printout of the SOP. Each participant was also attributed a random code (NXX for NT.115 instruments or PXX for NT.Pico instruments, respectively) so the automated analysis was anonymized.

### MST/TRIC measurements

NanoTemper is using the term MST (microscale thermophoresis) exclusively for the capillary-based Monolith instrument, while for the multiwell plate-based Dianthus, it is using the term TRIC (temperature-related intensity change) for the very early intensity changes formerly known as the T-Jump region in Monolith measurements. We would propose to use the term TRIC as a more general term applicable for the whole MST/TRIC time trace since the measured signal corresponds in both cases to a “temperature related intensity change”, stemming from various sources. A more detailed investigation of the time traces and the effects that can be observed is given in (López-Méndez 2021). Throughout this manuscript MST and TRIC are therefore used in conjunction to describe the experiment and its analysis, and MST is only used alone for expressions such as “MST Power”.

Each participant was provided a protocol/SOP to be followed for sample preparation and measurement (see supplementary material 4).

### Data analysis

Measurements were analyzed by the participants, from now on called user analysis, according to their usual practice. The *K*_D_ values as well as additional information about the measurements (e.g. user estimated noise/errors, how the data were analyzed) were collected using a standardized form (see supplementary material 4).

All measurements were also centrally analyzed using the MO.Affinity Analysis 2.3 software provided by NanoTemper (MOAA) as well as the PALMIST 1.5.6 software provided by Chad Brautigam (Scheuermann 2016).

### Extracting *F*_norm_

In the centralized data analysis, a conservative definition of outliers was used: only data points where either absolute fluorescence or capillary scan shape showed irregularities or MST/TRIC traces showed bleaching and/or artifacts from aggregation (‘bumps’) were defined as outliers. The procedure below was followed to extract *F*_norm_ values and subsequently fit the binding curve.

**Fig. 1:**
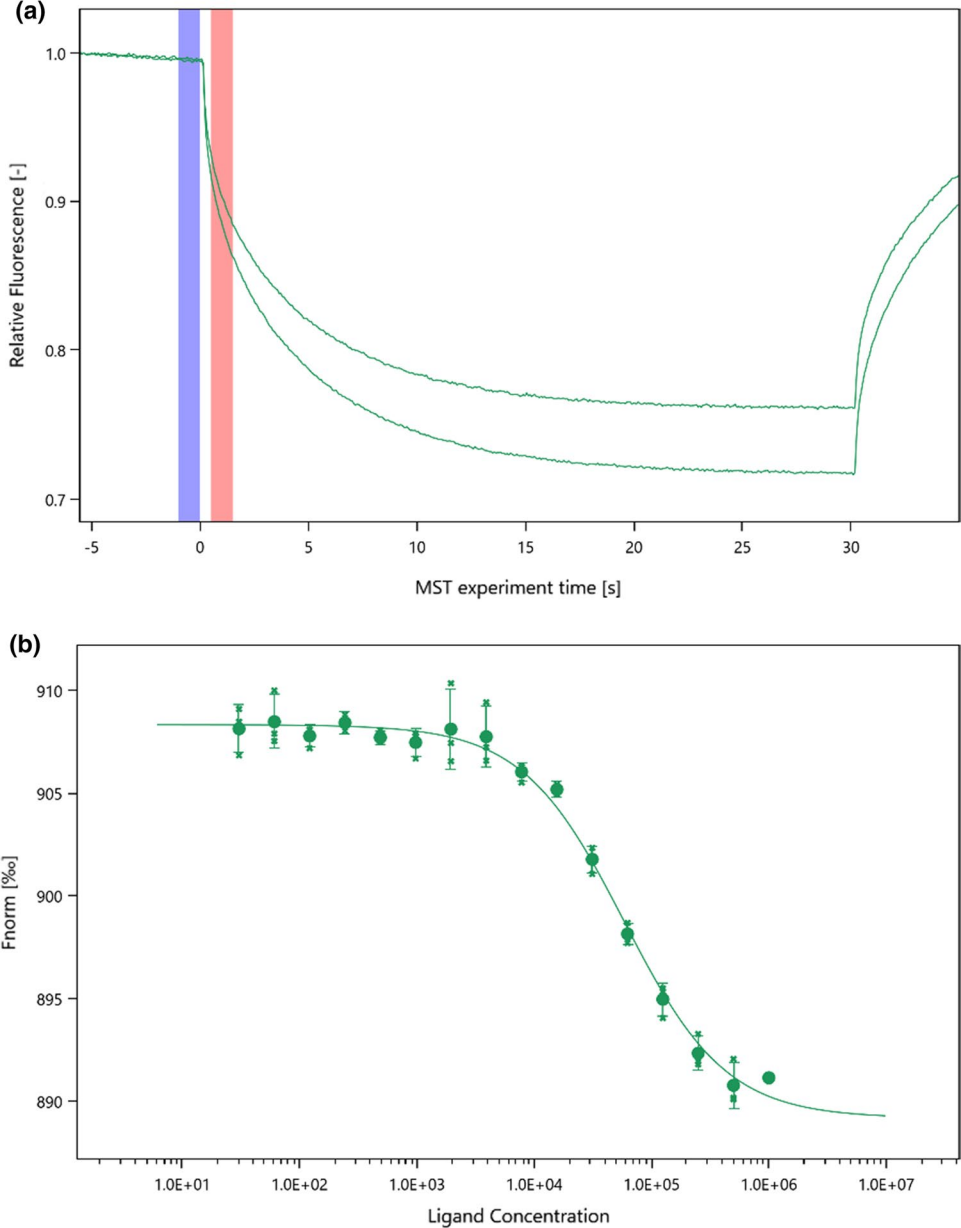
**a** Representative MST/TRIC time trace of 25 nM labeled lysozyme in PBS + without (upper curve) and with 1 mM NAG3 (lower curve). Cold (blue) and hot (red) regions as defined in central data analysis to calculate *F*_norm_. **b** Representative binding curve for the lysozyme–NAG3 interaction. Data of both figures are from instrument N06. Diagrams exported from MOAA

The T-Jump corresponds to the moment the IR laser is turned on and a rapid decrease in fluorescence intensity is present in the MST/TRIC time trace, this is defined as time 0. The cold region has been defined as the 1 s region just before the T-Jump. The hot region has been defined as 0.5–1.5 s after the T-Jump (see Fig. 1a).

*F*_norm_ is defined as the ratio between the average fluorescence in the hot region and the cold region.

1 $${F}_{\mathrm{norm}}=\frac{\langle {F}_{\mathrm{hot}}\rangle }{\langle {F}_{\mathrm{cold}}\rangle }$$

### Binding curve fitting

Binding curve data (Fig. 1b) were fitted using the mass action law as outlined in Baaske (2010), Scheuermann (2016) and in the supplementary information.

For different fluorescence intensities of the bound state *F*_AB_ in comparison to the unbound state *F*_A_, corrections to the linearity assumptions for *F*_norm_ need to be considered, assuming a fluorescence ratio between bound and unbound target $y=\frac{{F}_{\mathrm{AB}}}{{F}_{\mathrm{A}}}$.

Accounting for the different weighting of the *F*_norm_ signal stemming from the unbound and the bound state, respectively, the equation reads:

2 $${F}_{\mathrm{norm}}\left(X\right)=\frac{{F}_{\mathrm{norm}}(0)+X \left({F}_{\mathrm{norm}}(1)\bullet y-{F}_{\mathrm{norm}}(0)\right)}{1+X\left(y-1\right)}$$

$X$ being the bound fraction (between 0 and 1). For a fluorescence ratio of *y* = *1* this simplifies to supplementary equation (ii) which is normally used. Unfortunately, Eq. (2) has neither been implemented in MOAA nor in PALMIST. One could still carry out the fitting using other data analysis tools, but this was beyond the scope of this benchmark study. The fluorescence-corrected equation was discussed in the supplementary of (Baaske 2010) and is already implemented in NanoTemper’s Dianthus software.

### S/N

Measurement signal (*S*) for each triplicate data set has been defined as the amplitude of the binding signal derived from the fitting procedure.

3 $$S={F}_{\mathrm{norm}}\left(1\right)-{F}_{\mathrm{norm}}\left(0\right)$$

Measurement noise (*N*) has been defined as the average of *F*_norm_ standard deviations ${\sigma }_{i}$ of the replicates for each ligand concentration instance *c*_B,*i*_ (${\sigma }_{i}$ corresponding to the error bars seen in Fig. 1b).

4 $$N=\langle \sigma \rangle =\frac{1}{n}\sum_{{c}_{\mathrm{B},i}}^{{c}_{\mathrm{B},n}}{\sigma }_{i}$$

Notably, no fitting model is implied here, just the reproducibility of the *F*_norm_ readout values is used.

A signal-to-noise ratio defined as *S/N*, reports on the reproducibility of a measurement for a given instrument and participant. *S/N* does not decrease for an increasing number of replicates and can give an estimate of how many replicates will actually be needed to achieve a certain accuracy of the results.

All the equations above are in-line with what was used in Baaske (2010) and are in contrast to how noise is reported in the MO.AffinityAnalysis software, as can be read in the respective description of the fitting procedure. MOAA noise (*N′*) corresponds to the standard deviation of the difference between averaged experimental data and fitted data.

5 $$N^{\prime}{\text{ = }}\sqrt {\frac{{\text{1}}}{{n - {\text{1}}}}\sum\limits_{{{\text{c}}_{{{\text{B}},{\text{i}}}} }}^{{\text{n}}} {\left( {\langle {\text{F}}_{{{\text{normexp}},i}} \rangle - {\text{F}}_{{{\text{normfit}},i}} } \right)^{{\text{2}}} } }$$

*N′* therefore actually reports on the goodness of the fit of the average data to an ideal binding curve, and while this is a valid approach for combining replicate data, it does not report on the variability of the replicates that we wish to investigate. For instance, if the averages of the replicate values align perfectly to the fit, *N′* is expected to go to 0 even if the standard deviations for the replicates are finite.

## Results and discussion

### Hardware variability

**Fig. 2:**
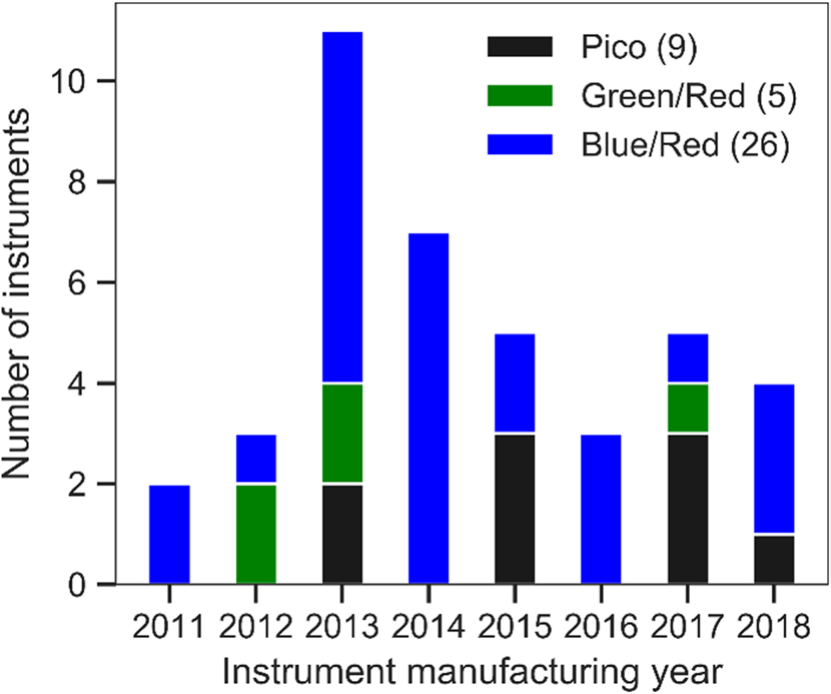
Number of NT.Pico (black) and NT.115 Monolith instruments with different filter sets (red/green and red/blue) per year of manufacture (encoded in the instrument serial number)

Overall, 31 NT.115 and 9 NT.Pico instruments were used in the benchmark study. The years of instrument manufacture and the installed filter sets are shown in Fig. 2.

**Fig. 3:**
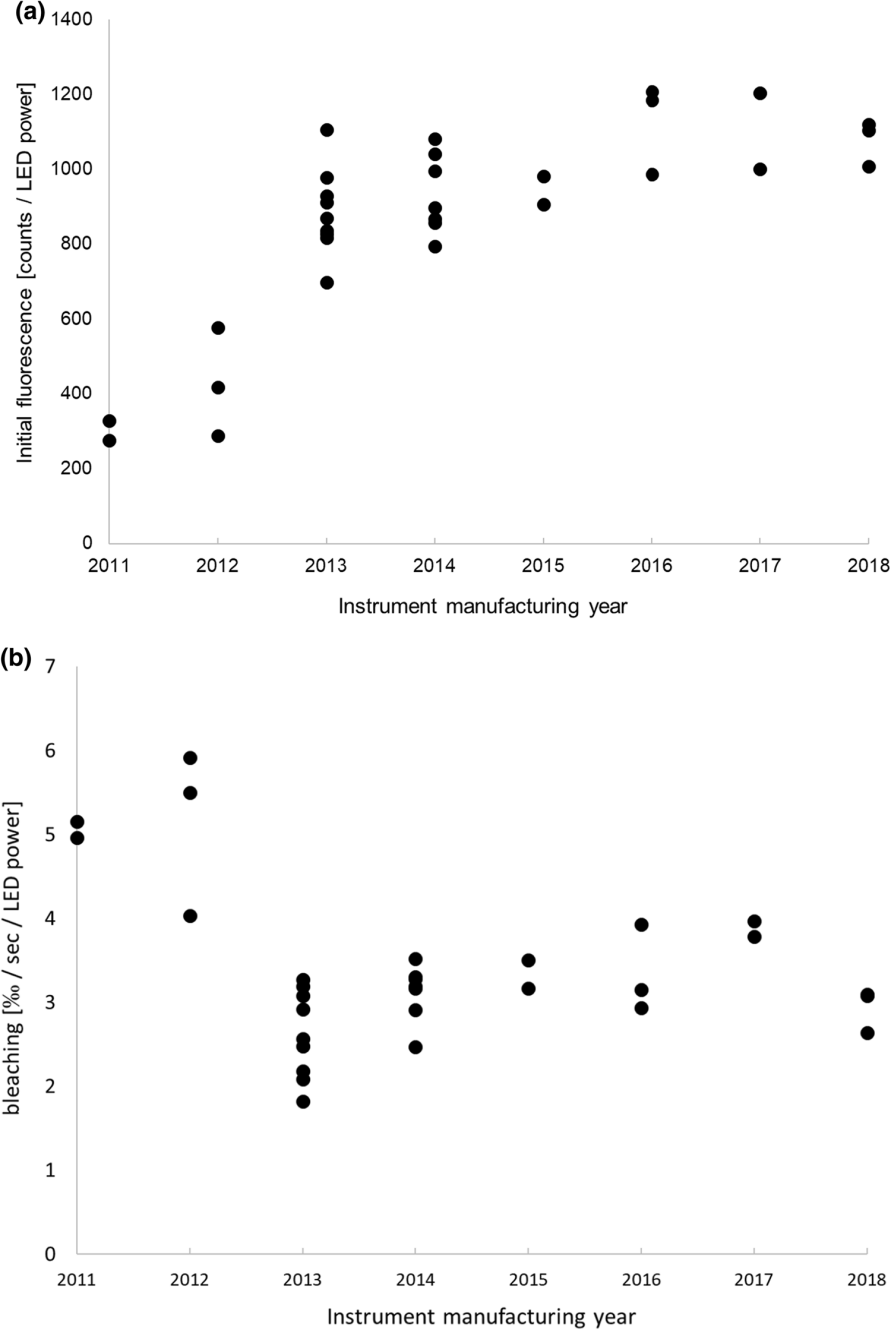
**a** Absolute fluorescence counts per LED power at the start of the MST/TRIC time trace and **b** negative slope during the first 5 s (bleaching) of the MST/TRIC time trace for a 25 nM NHS-RED 2^nd^ generation dye for each NT.115 instrument versus their manufacturing date

The hardware variability was tested using a solution of 25 nM RED-NHS, 2^nd^ generation dye in PBS + . Absolute fluorescence counts per LED power as well as the bleaching effect (slope of the MST/TRIC time trace within the first few seconds) per second and LED power showed significant differences for NT.115 instrumentation prior to 2013 (Fig. 3a, b) but not in NT.Pico instruments (data not shown) which can be explained by a change in hardware by NanoTemper for more recent instruments (different detectors used from 2013 onwards, oral communication).

Typical *F*_norm_ noise *N* within an individual instrument is about 0.8–1.7‰. Comparing *F*_norm_ values derived for low, medium and high MST power (which corresponds to 20%, 40% and 60% MST power, respectively, as is implemented in MOAA) of different instruments as shown in supplementary Fig. 1a, yield a considerable standard deviation of about 15–20‰. This is, however, not linked to instrument age, because no obvious instrument manufacturing year dependence can be seen for this distribution (see supplementary Fig. 1b).

Although no significant correlation between *F*_norm_ values and reported lysozyme—NAG3 *K*_*D*_ values could be seen (see supplementary Fig. 2), large variations in *F*_norm_ could pose a difficulty when combining and comparing raw data of measurements performed on different instruments. The variation of *F*_norm_ values between instruments could stem from, e.g., different optical properties and geometries as well as from different heating powers of the respective IR lasers.

During production, NanoTemper calibrates the Monolith for similar *F*_norm_ values to minimize those variabilities. However, after the significant *F*_norm_ variability of the instruments has been identified within the scope of this benchmark (see supplementary Fig. 1), NanoTemper further revised their calibration procedures (see supplementary material 4).

### *K*_D_ variability

The variability of *K*_*D*_ values generated by NanoTemper Monolith instruments has been assessed using RED-NHS 2^nd^ generation dye labeled lysozyme as a target and the small trisaccharide NAG3 as a ligand. This is a stable and facile standard system applicable to the widest range of instruments.

Comparison of *K*_*D*_ values for unlabeled lysozyme to NAG3 of about 4 µM (using the NT.LabelFree approach) to values generated by ITC (6.5–8.5 µM) showed good agreement (see supplementary Fig. 3). However, upon labeling of lysozyme, the results showed a change in *K*_D_ depending on the respective dye and buffer system used (10.0–97.6 µM) (see supplementary Table 1). Consequently, *K*_D_ values measured in this benchmark study for the labeled lysozyme–NAG3 interaction cannot be directly compared with *K*_D_ values generated by MST/TRIC or other techniques with unlabeled lysozyme.

**Fig. 4:**
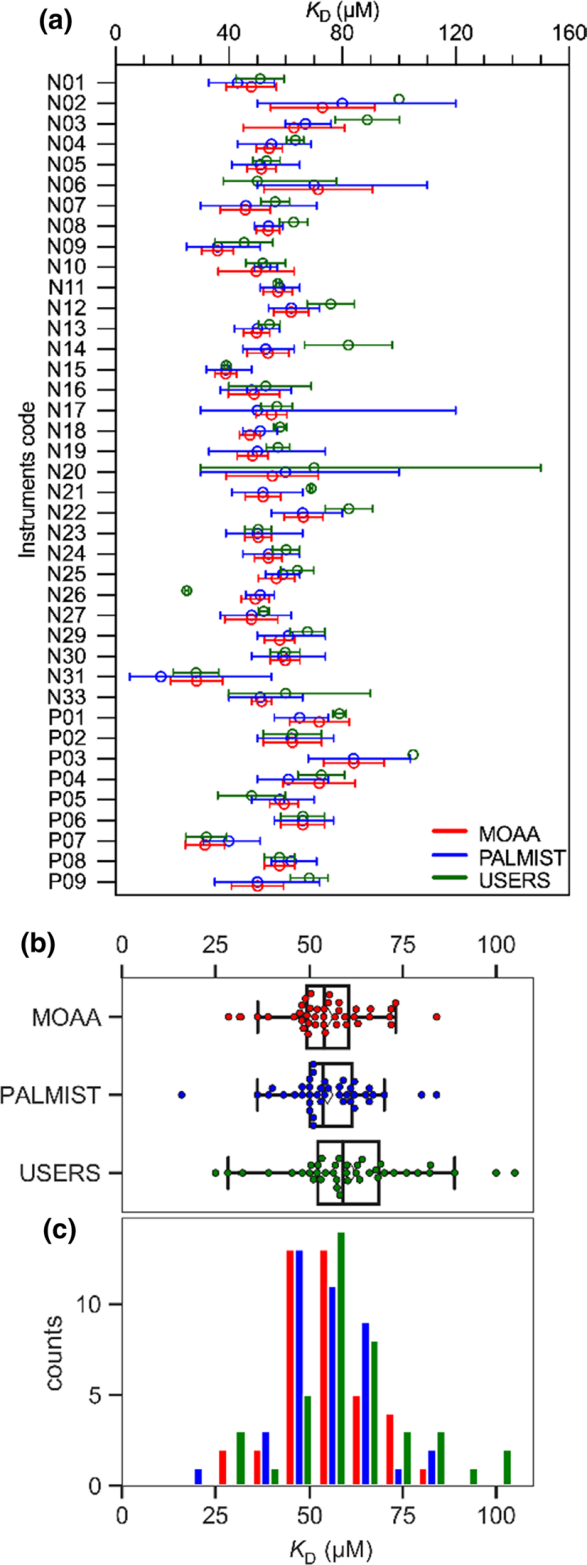
**a** The experimentally determined *K*_D_ values together with the 68.3% confidence intervals from central analysis (MOAA and PALMIST) and estimated errors reported by participants in the benchmark study (USERS) for each Monolith NT.115 (N) and NT.Pico (P) instrument. For N02 no errors or confidence intervals were reported by the participant. **b** Swarm plots superposed on standard box-and-whisker plots. The box covers the inter-quartile range (IQR), the vertical line in the box is the median, and the range represented by the “whiskers” extends from Q1 – (1.5 × IQR) to Q3 + (1.5 × IQR). Means are marked with white diamonds. **c** A histogram of the data pooled in 10 µM steps

The resulting values for the *K*_D_ of the labeled lysozyme–NAG3 interaction as well as their 68.3% confidence intervals (from the covariance matrix for MO.Affinity Analysis and from the error-surface projection for PALMIST) and the estimated errors (from the users) are shown in Fig. 4a. Each instrument has been given a code (N for NT.115 and P for NT.Pico instruments) to anonymize the respective measurement and user’s data analysis. A boxplot (Fig. 4b) and a histogram (Fig. 4c) of the combined data emphasize the distribution.

As shown in Fig. 4c, the distributions of results from all three analysis methods are substantially overlapped. In Table 1, the average *K*_D_, standard deviation σ and standard error of mean ${\sigma }_{\langle {K}_{\mathrm{D}}\rangle }$ of the combined data from all instruments are summarized and very similar results for both central analysis types (MOAA and PALMIST) can be observed. The mean *K*_D_ from users’ analysis is, however, substantially outside the standard error generated by the central analysis but still within the standard deviation.

**Table 1:** Lysozyme–NAG3 interaction results and statistic parameters

Analysis type TRIC	Mean $\langle {K}_{D}\rangle$ [µM]	Standard deviation σ [µM]	Standard error of mean ${\sigma }_{\langle {K}_{\mathrm{D}}\rangle }$[µM]	Relative standard deviation ${c}_{V}$	Average signal-to-noise ratio $\langle S/N\rangle$
MOAA (central)	54.8	11.0	1.7	0.201	29.7
PALMIST (central)	54.8	11.6	1.8	0.211	24.2
USERS (individual)	60.9	16.6	2.6	0.273	–^a^

^a^Noise parameters reported by the users showed large differences and did not follow the definition in the methods section

The relative standard deviation of the extracted *K*_D_ values, also known as the coefficient of variation

6 $${c}_{V}=\frac{\sigma }{\langle {K}_{\mathrm{D}}\rangle }$$

is approximately 20% for the centralized analysis while it is about 27% for the individual analysis for the users.

**Fig. 5:**
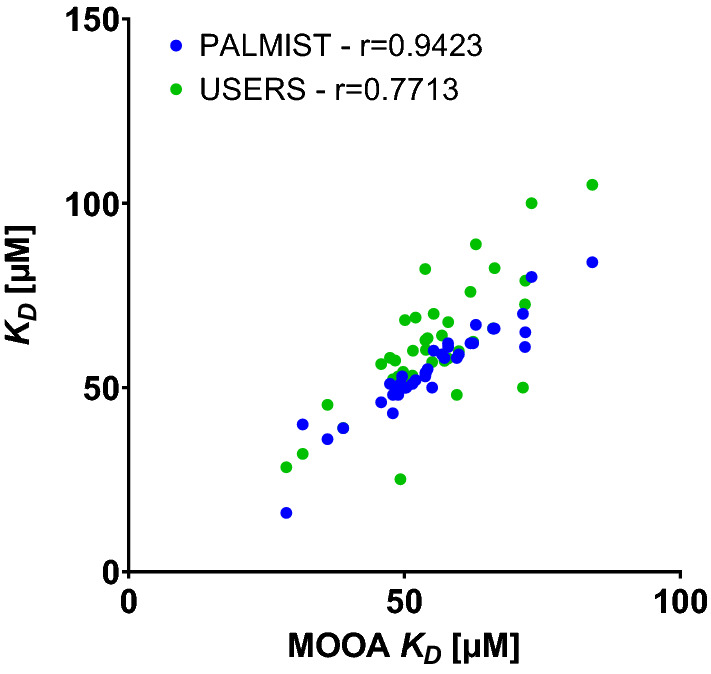
Comparison of lysozyme—NAG3 *K*_D_s extracted for each individual instrument for different analysis tools (blue, PALMIST vs MOAA) and individual versus central analysis (green, users vs MOAA) and respective Pearson r

To emphasize where the differences, especially between user specific and central analysis originate from, the *K*_*D*_s extracted for each dataset from centralized PALMIST and from individual users’ analysis are plotted against the *K*_D_ values extracted from central MOAA analysis in Fig. 5.

To quantify the variation in results stemming from the use of different analysis tools vs. individual analysis strategies in comparison to variation between experiments, we define the relative standard deviation of differences of *K*_*D*_ ${c}_{V,\Delta {K}_{\mathrm{D}}}$ from the different analyses applied to individual experimental datasets, i, using the central MOAA analysis as the reference.

7 $${c}_{V,\Delta {K}_{\mathrm{D}},\mathrm{PALMIST}}=\frac{{\sigma }_{\Delta {K}_{\mathrm{D}}, \mathrm{PM}}}{\langle {K}_{\mathrm{D}}\rangle }=\frac{1}{\langle {K}_{\mathrm{D}}\rangle }\sqrt{\frac{1}{n-1}\sum_{i}^{n}{\left({K}_{\mathrm{D},\mathrm{ PALMIST}}-{K}_{\mathrm{D},\mathrm{ MOAA}}\right)}^{2}}$$

${c}_{V,\Delta {K}_{\mathrm{D},\mathrm{ PALMIST}}}$, arising from identical central analysis strategies but different analysis tools, is about 7%, while ${c}_{V,\Delta {K}_{\mathrm{D},\mathrm{ Users}}}$, arising from the difference when comparing individual analysis strategies applied by users to central analysis in MOAA, is about 23%.

This means that variation in results arising from different analysis tools (7%) is on average a minor effect compared to that from different analysis strategies (23%), which is itself of similar magnitude to the variation between experiments from different instruments/participants (20% using identical strategies and tools).

### Software differences

Certain differences in the raw data processing for PALMIST and MOAA have been observed which are assumed to be the reason for the 7% variability between using different analysis tools.

In MO.AffinityAnalysis, the average *F*_norm_ of each triplicate experiment were used for fitting, while in PALMIST all individual *F*_norm_ replicates of an experiment were used for global fitting (which is preferable if there are different number of replicates per data point due to outliers).

Subtle but significant differences between MOAA and PALMIST treated dataset (e.g. for signal-to-noise ratios) revealed two other effects.

First, MOAA is not directly averaging replicate measurements but is beforehand applying an absolute shift to each set of replicate *F*_norm_ values to minimize the noise. This results in an artificially lowered noise (both *N* and *N′*) and also *n*—1 added parameters for the analysis, n being the replicate number. It was implemented by NanoTemper due to the need for averaging data from different instruments and to compensate for the variability of absolute *F*_norm_ values generated by different instruments (see supplementary material 4).

**Fig. 6:**
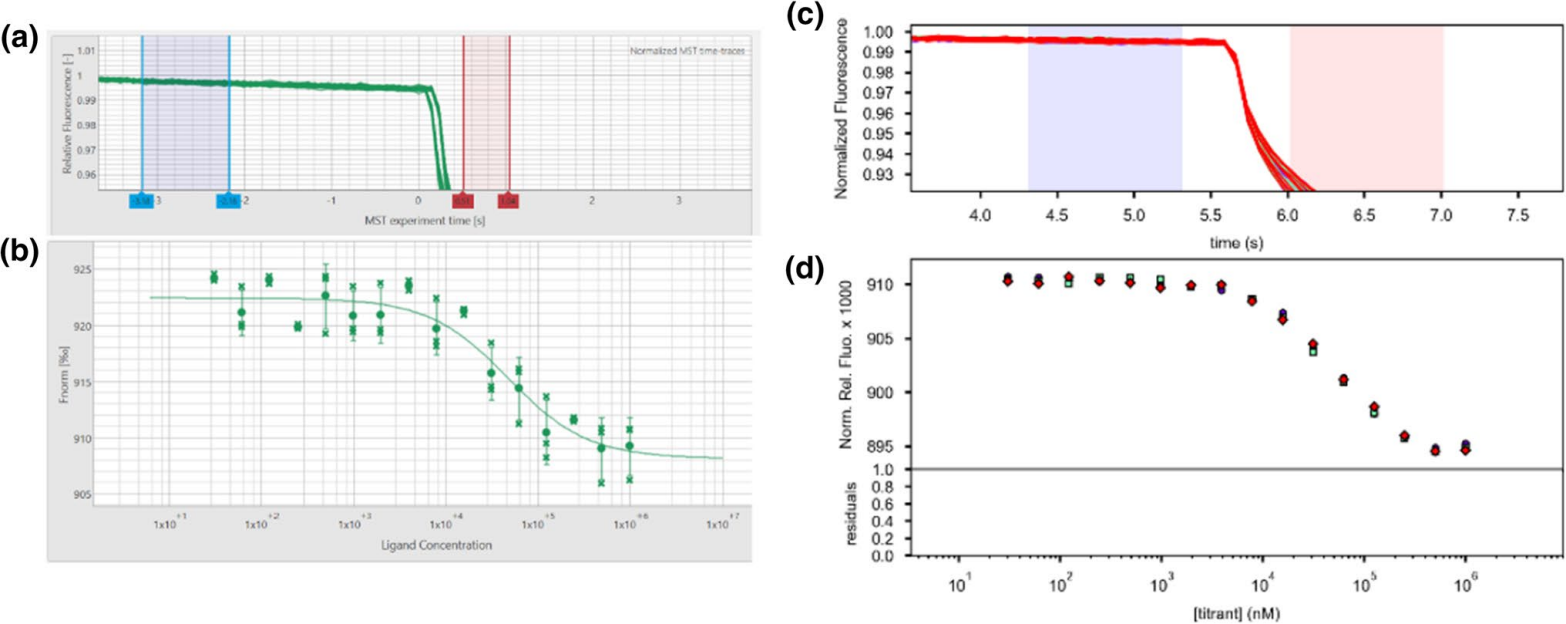
Time offset of the T-Jump position not properly accounted for in MOAA (**a**) and resulting higher noises (**b**) compared to properly accounted for T-Jump time in PALMIST (**c**) and (**d**) for dataset N10. Diagrams are screenshots of MOAA and PALMIST, respectively

Second, MOAA is not correctly accounting for time shifts in data generated by certain NT.Control software versions. Therefore, the T-Jump region is sometimes shifted by one datapoint (~ 75 ms), as can be seen in Fig. 6.

This offset did not have a large influence on the value of *K*_D_, but only on the error of *K*_D_ (as can be seen for dataset N10 in Fig. 4a). To further examine the reliability of the two different data analysis tools, one can count how many times the mean $\langle {K}_{\mathrm{D}}\rangle$ of all experiments (54.8 µM) was within the predicted 68.3% confidence interval for each instrument (shown in Fig. 4a). If the confidence interval estimates are accurate this proportion should be close to 68.3%. For PALMIST in 77.5% of all case $\langle {K}_{\mathrm{D}}\rangle$ fell within the interval, while for MOAA, it was only 62.5% (also see Table 5).

### Analysis strategy differences

To find the sources of the differences between the central and the individual data analysis, we investigated the different analysis strategies applied by users.

The individual freedom of how to analyze the data is limited mainly to two aspects, one being the way how outliers are chosen, although this was found to be a minor issue during this benchmark study. Another opportunity for individualism is where to choose the hot region that is going to be used for *F*_norm_ calculation and therefore for subsequent data analysis.

**Fig. 7:**
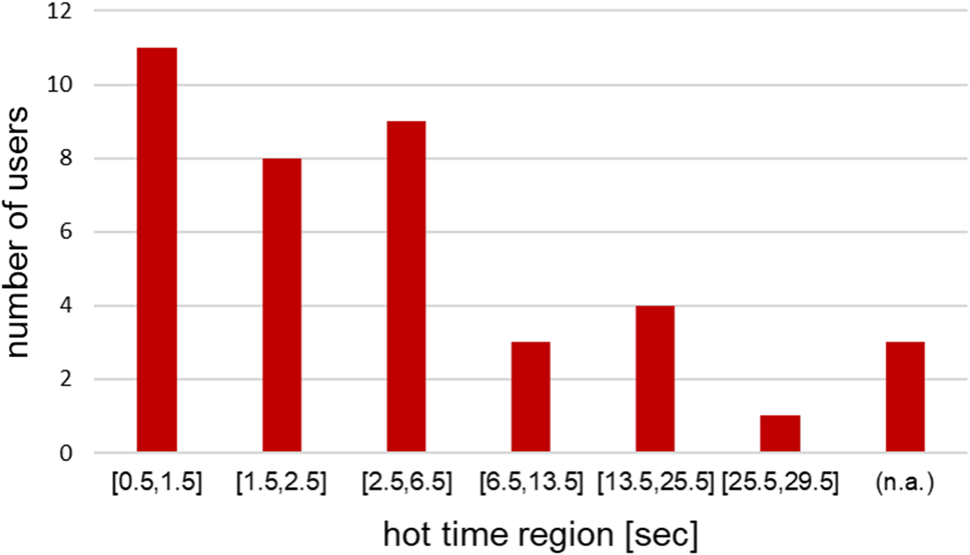
Number of users choosing the hot time region within the respective time interval along the MST/TRIC time trace. 0 s corresponding to the T-Jump position, (n.a.) if no hot time was reported

It is strongly recommended to analyze the early part of the MST/TRIC time trace to minimize any temperature dependent artifacts that could possibly arise (López-Méndez et al. 2021). Several participants followed this guideline, as we did with the central analysis procedure, while others analyzed later parts of the MST/TRIC time trace (as it was the recommended best practice several years ago). The frequencies of the chosen hot time regions for analysis in the MST/TRIC time trace are shown in Fig. 7 (0 s corresponding to the T-Jump, see Fig. 1a).

**Fig. 8:**
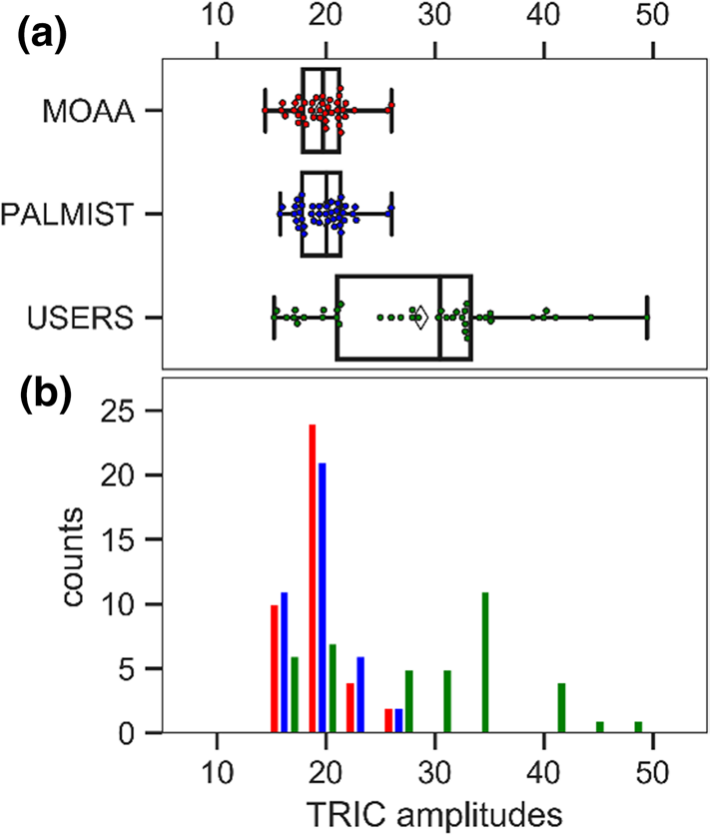
**a** Swarm plots of individual binding amplitude signals in ‰ for lysozyme–NAG3 interaction superposed on standard box-and-whisker plots for centralized MOAA and PALMIST analysis as well as for individual users’ analysis. The box covers the inter-quartile range (IQR), the vertical line in the box is the median, and the range represented by the “whiskers” extends from Q1 – (1.5 × IQR) to Q3 + (1.5 × IQR). Means are marked with white diamonds. **b** A histogram of the data pooled in 3 ‰ steps

It seems that utilizing different regions along the MST/TRIC time trace not only resulted in large variations in the amplitude of the signal (as can be seen in Fig. 8a, b), but also in a greater variability and a shift in mean *K*_D_ (Table 1). Such a shift has also been reported in Scheuermann (2016).

As an additional source of variability in individual analysis, two different measurement tools (NT Control and MO.Control) as well as three different analysis tools (NT Analysis, MO.Affinity Analysis and PALMIST), each of them also present in different software versions with different presets for analysis, were used by the participants, as summarized in Table 2.

**Table 2:** Frequency of different measurement software (NT Control and MO.Control) as well as software used for analysis (NT Analysis, MOAA, PALMIST) chosen by users

	NT analysis	MOAA	PALMIST
NT Control	2	13	6
MO.Control	0	21	1

The sum is bigger than 40 since some users reported results from different analysis tools at the same time

### Dataset variability between instruments

Even disregarding the variation arising from different software tools and from individual analysis strategies (mainly the chosen times where the “hot” fluorescence was measured), a relative standard deviation of about 20% between datasets from different instruments is found (Table 1). The average noise *N* of *F*_norm_ as defined in the methods section for a triplicate measurement of the lysozyme–NAG3 interaction in the Monolith instrument was about 1‰ while the average signal-to-noise ratio S/N of all datasets was about 24–30 (see Table 1) but ranging from single digit numbers to more than 70. Clearly a higher variability in *K*_D_ can be observed for measurements with lower S/N ratios (as is shown in supplementary Fig. 4).

MOAA generally showed slightly higher S/N ratios than PALMIST since the shift correction applied to replicates (see software differences above) lowered the calculated noise.

To further investigate the influence of the signal-to-noise ratio on the observed variation of *K*_D_ another test system was measured.

### *K*_D_ variability—additional challenges

It was desirable to test a more challenging interaction system to provide further insight into sources for measurement variability as well as to identify potential systematic errors. Therefore, labeled lysozyme as a target and a nanobody (NB) as a ligand were employed as a test system.

**Fig. 9:**
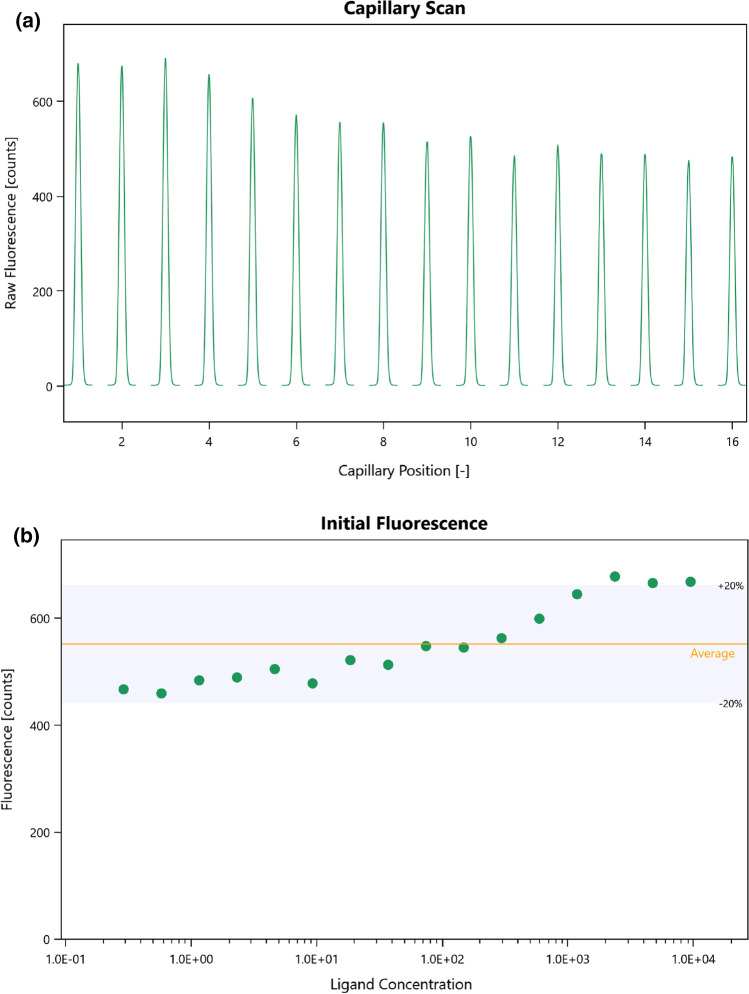
Representative capillary scans for the lysozyme–NB interaction (**a**) and absolute fluorescence values for each ligand concentration (**b**) as shown in the MOAA software. The absolute fluorescence average and ± 20% range are shown in MOAA to emphasize the variation. Figures are screenshots from MOAA

In addition to a TRIC binding signal along the MST/TRIC time trace, this interaction showed a change in absolute fluorescence upon binding, as can already be seen in the capillary scan (see Fig. 9a).

For a fluorescence change upon binding that is larger than 20% of the absolute signal the current recommendation (best practice) is to analyze the initial fluorescence signal (Fig. 9b) instead of the TRIC signal, once it has been confirmed that the fluorescent change is due to the interaction (see for instance *Initial Fluorescence* and *SD-Test* in Nanopedia 2018 or *SD-Test* in Nanopedia 2020). The reason for this is that the current equations [supplementary equation (i) and (ii)] employed for fitting the TRIC signal (both in MOAA and PALMIST) assume identical weighting of the bound and unbound state signal, which is not the case if the bound and unbound state differ in absolute fluorescence (see methods section). The fluorescence variation corrected Eq. (2) accounting for different weights of the bound and unbound state can be found in the methods section and in Baaske (2010).

**Fig. 10:**
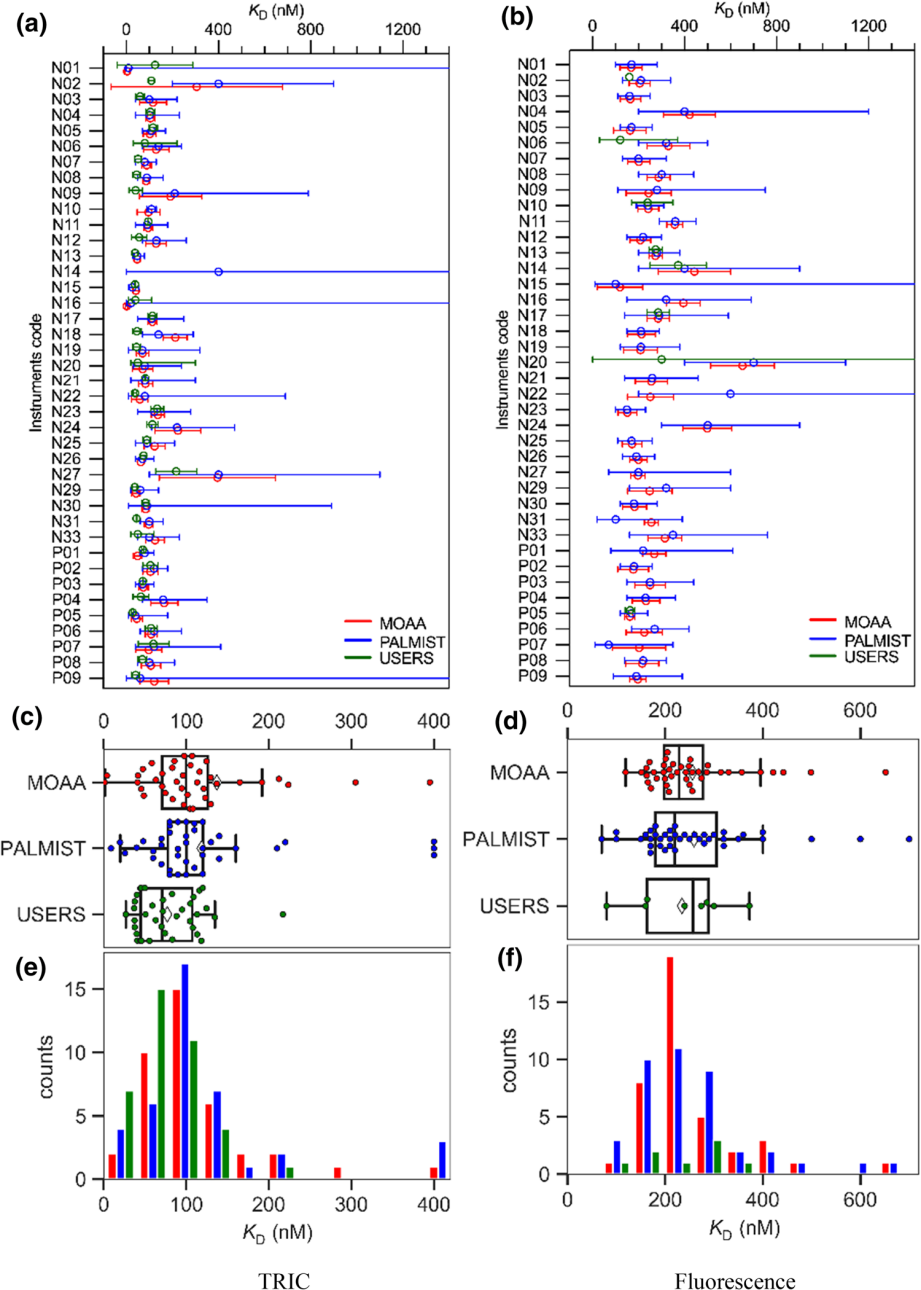
The experimentally determined *K*_D_ values and 68.3% confidence intervals (CI) for lysozyme–NB interaction measurements from central analysis (MOAA and PALMIST) and estimated errors reported by users (USERS) for each Monolith NT.115 (N) and NT.Pico (P) instrument for TRIC analysis (**a**) and absolute fluorescence analysis (**b**). Upper CI limits generated by PALMIST for the fluorescence analysis of N15 and for the TRIC analysis of N01, N14, N16 and P09 were undeterminable as well as the lower CI limits for the TRIC analysis of N01 and N16. Upper PALMIST CI limit for the fluorescence analysis of N22 is 1800 nM. **c** and **d**, swarm plots superposed on standard box-and-whisker plots for *K*_*D*_ values in **a** and **b** respectively. The box covers the inter-quartile range (IQR), the vertical line in the box is the median, and the range represented by the “whiskers” extends from Q1 – (1.5 × IQR) to Q3 + (1.5 × IQR). Means are marked with white diamonds. **e** and **f** show histograms of the data in (**c**) and **d** respectively, pooled in 50 nM steps

In the case of lysozyme–NB interaction, the relative change in the fluorescence is on average 36% but despite this only 8 out of 40 participants noticed and responded to the change in fluorescence and analyzed the data accordingly. For comparison, both the TRIC signal of the lysozyme–NB interaction as well as the absolute fluorescence signal were analyzed using supplementary equation (ii), although the TRIC signal should have been more correctly analyzed using Eq. (2). The distribution of *K*_D_ values generated for TRIC analysis and for absolute fluorescence analysis is shown in Fig. 10, respectively. The results for the MOAA TRIC analysis of N14 had to be removed because it gave a value of 1170 nM, although it did not show typical signs of an outlier. Including this value would have had a disproportionate effect on the statistical results and the conclusion thereof.

Broader distributions with standard deviation of similar magnitude to their mean value are observed for this interaction as summarized in Tables 3 and 4. This is most likely the case due to the much lower signal-to-noise ratio for this interaction of about 5–9 for TRIC and 10–15 for the fluorescence analysis.

**Table 3:** Lysozyme–NB interaction results and statistic parameters for TRIC analysis

Analysis type TRIC	Mean $\langle {K}_{\mathrm{D}}\rangle$ [nM]	Standard deviation σ [nM]	Standard error of mean ${\sigma }_{\langle {K}_{\mathrm{D}}\rangle }$ [nM]	Relative standard deviation ${c}_{V}$	Average signal-to-noise ratio $\langle S/N\rangle$
MOAA (central)	111	74	12	0.667	8.6
PALMIST (central)	119	90	14	0.763	5.2
USERS^a^ (individual)	76	38	10	0.501	–^b^

^a^38 out of 40 reported values

^b^Noise parameters reported by users showed large differences and did not follow the definition in the methods section

**Table 4:** Lysozyme–NB interaction results and statistic parameters for fluorescence analysis

Analysis type fluorescence	Mean $\langle {K}_{\mathrm{D}}\rangle$ [nM]	Standard deviation σ [nM]	Standard error of mean ${\sigma }_{\langle {K}_{\mathrm{D}}\rangle }$ [nM]	Relative standard deviation ${c}_{V}$	Average signal-to-noise ratio $\langle S/N\rangle$
MOAA (central)	256	104	16	0.405	15.1
PALMIST (central)	259	125	20	0.482	9.8
USERS^a^ (individual)	272	64	26	0.235	–^b^

^a^8 out of 40 reported values

^b^Noise parameters reported by users showed large differences and did not follow the definition in the methods section

The confidence intervals for each individual dataset of the lysozyme–NB interaction are much larger in comparison to the lysozyme–NAG3 interaction and in some cases, even reach physically nonsensical negative values for intervals deduced by the covariance matrix, as can be seen in Fig. 10a, b. This is because the covariance matrix is not necessarily correctly predicting the confidence interval of nonlinear fits with lower S/N, especially when the symmetry assumption for the confidence interval is not valid.

The mean *K*_D_ values from the central analyses of the TRIC data agree within the standard error of mean, as do both the analyses of the fluorescence data. User data do again show a shift in mean *K*_D_ and, in this case, a smaller standard deviation indicating an effect of individual preference during data analysis. However, there is a significant difference between the *K*_D_ from the TRIC analysis of about 111 nM and the *K*_D_ from the fluorescence analysis of about 256 nM. This could be partially explained by the fact that the bound state will show a stronger signal (on average by about 36%) than the unbound state and therefore the fraction of bound state is overestimated in the TRIC analysis. Another reason for this difference in *K*_D_ can be found in several TRIC interaction curves that show a slight change in the baseline for higher ligand concentrations (Supplementary Fig. 5). The reason for this deviation from an ideal 1:1 binding curve has not yet been elucidated, but similar behavior has been reported in Scheuermann (2016). However, the measured binding curve for the fluorescence analysis corresponds more closely to the expected shape of a 1:1 binding curve (Supplementary Fig. 6).

**Fig. 11:**
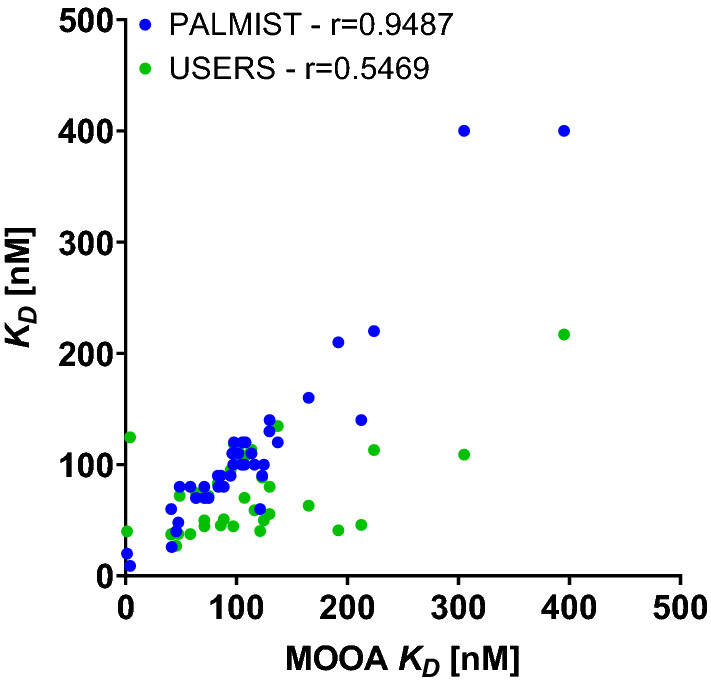
Comparison of *K*_D_ values extracted for each individual instrument for different analysis tools (blue, PALMIST vs MOAA) and individual versus central analysis (green, users vs MOAA) for lysozyme–NB interaction using TRIC analysis and respective Pearson r

Again, the consistency of the two central analyses of the TRIC data extends beyond agreement of their means. Datasets which have a small *K*_D_ analyzed in MOAA also have a small K_D_ in PALMIST (Fig. 11). The same is true for the analysis of the fluorescence data (not shown). This could for instance be stemming from systematic pipetting errors during the preparation of the concentration series resulting to certain instruments generally showing “lower” *K*_D_ values than others, or it could be simply a stochastic effect of the measurement stemming from the respective signal-to-noise and the low number of replicates (triplicate) measured for each instrument.

**Fig. 12:**
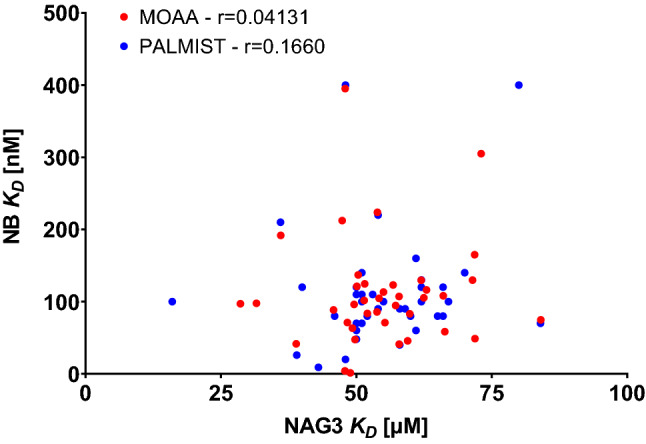
For each individual instrument *K*_D_s for the lysozyme–NB interaction are compared to the *K*_D_s for the lysozyme–NAG3 interaction

To address whether there is a systematic error involved in instruments showing lower or higher *K*_D_s or if this is just a stochastic phenomenon, individual instruments *K*_D_s for the lysozyme–NAG3 interaction are compared to *K*_D_s for the lysozyme–NB interaction in Fig. 12.

No correlation between the *K*_D_s can be found and therefore the observed variability appears to be stochastic.

**Fig. 13:**
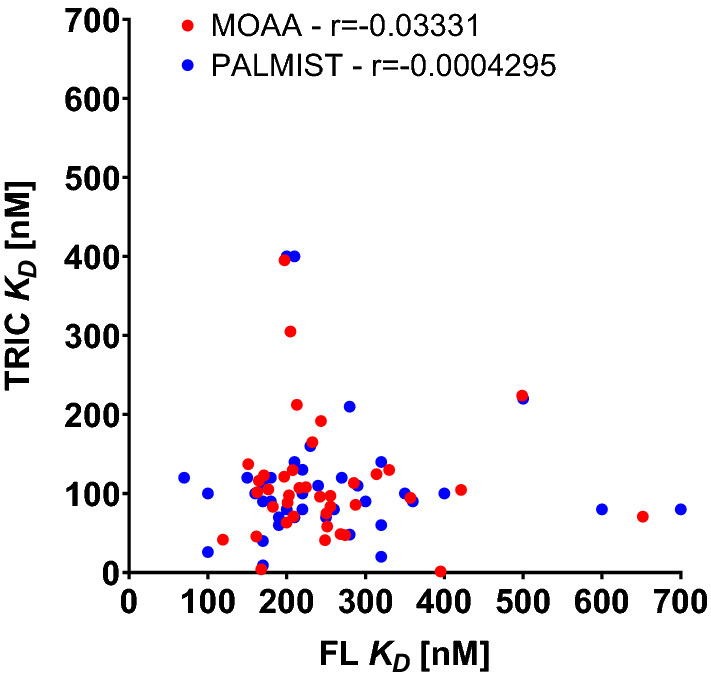
*K*_D_ values for TRIC analysis of lysozyme–NB interaction compared to *K*_D_ values from fluorescence analysis for each instrument dataset

Moreover, the *K*_D_ values for the lysozyme–nanobody interaction generated by TRIC and by fluorescent analysis of the same datasets are not correlated either as can be seen in Fig. 13, so systematic ligand concentration errors from pipetting can be excluded.

Low *K*_D_ values from the TRIC signal do not necessarily correspond to low *K*_D_ values from the fluorescence signal. On the contrary, the independent source of information stemming from the TRIC and the fluorescence signal could provide a way to increase the reliability for *K*_D_ values for experiments where, upon binding, both the TRIC signal (shown by almost all types of molecular interactions) and the fluorescence signal change (also visible in several molecular interactions) are present, provided the authenticity of the fluorescence signal has been ensured e.g. by an *SD-Test* (Nanopedia 2018 or Nanopedia 2020) or other negative controls. A global fitting procedure taking into account both the fluorescence signals [supplementary equation (ii)] as well as the (fluorescence weighted) TRIC signals (Eq. (2)) would increase the dataset size typically 16 datapoints to 32 while only increasing the free parameters of the fitting model from 3 (*K*_D_, *F*_norm_ unbound, *F*_norm_ bound) to 5 (*K*_D_, *F*_norm_ unbound, *F*_norm_ bound, *F* unbound, *F* bound).

### Quantifying expected accuracies in Monolith measurements

The relative standard deviations ${c}_{V}$ of the *K*_D_s from the central data analysis show a reciprocal relation to the respective average signal-to-noise ratios of the raw data $\langle S/N\rangle$ (Tables 1, 3 and 4).

This is of course to be expected as increased measurement noise naturally leads to greater variation in fitted parameters. However, quantifying this dependence can help finding more suitable experiment design strategies [as shown in Wang et al. (2013)].

For the Monolith instruments in this benchmark study a relation of

8 $${c}_{V}\sim \frac{5}{\langle S/N\rangle }$$

for the coefficient of variation of *K*_D_ is found, where each individual estimate of *K*_D_ is derived from triplicates (*n* = 3). Equation (8) is approximately true for both TRIC and Fluorescence experiments in the central analyses for either PALMIST or MOAA (for $\langle S/N\rangle$ greater than 5). This means we can estimate the expected relative variation for other cases. For a single replicate instead of triplicate, we can estimate the relative standard deviation to be approximately $\sqrt{3}$ times bigger ($5\bullet \sqrt{3}\sim 9$) and thus, an expected relative standard error of the mean value for n replicates as

9 $$\frac{{\sigma }_{\langle {K}_{\mathrm{D}}\rangle }}{\langle {K}_{\mathrm{D}}\rangle }\sim \frac{9/\sqrt{n} }{\langle S/N\rangle }$$

One can predict the relative standard error of *K*_D_ of a typical triplicate measurement (*n* = 3) for a typical experimental scenario with a *S*/*N* of 15 to be ~ 35%. Or the other way around, if one wants to reach an uncertainty in *K*_D_ of about 20% for the same S/N, than at least 9 replicates will be needed. In practice, it would be necessary to estimate the S/N from at least triplicate experiments for the particular interaction and concentrations used.

### Comparison of Monolith results to other techniques

The scope of this benchmark was mainly to quantify variabilities within the Monolith measurements but obviously the comparison to other techniques like ITC and SPR is of great interest. As already mentioned, the lysozyme–NAG3 interaction is modified upon labeling of the lysozyme. However, this appears to be less of a concern for the lysozyme–NB interaction since the *K*_*D*_ agrees more closely with the results from other techniques.

The *K*_D_ of the nanobody–lysozyme interaction was measured to be 135 ± 35 nM by SPR and 103 ± 15 nM by ITC at 37 °C (Birchenough 2021) for unlabeled lysozyme, which is similar to both TRIC and fluorescence analysis within the experimental uncertainty (Table 3 and 4). This nanobody has a large CDR3 loop which experiences an extensive conformational rearrangement of the loop upon binding. This could contribute to the fluorescence change that is observed upon binding, which is not observed with other lysozyme nanobodies with shorter loops (Birchenough 2021).

## Conclusions

Although the absolute sensitivity of the different instruments is highly variable due to hardware detector changes and the hardware differences between the NT.115 and the NT.Pico Monoliths (Fig. 3), and also a significant variability in *F*_norm_ values at similar MST power (supplementary Fig. 1) is observed, the generated *K*_D_ values for a stable test system (lysozyme–NAG3) with a signal-to-noise ratio of about 24–30 agree very well. A relative standard deviation of *K*_*D*_ across all instruments of about 20%, when data are analyzed using a common strategy, is an outstanding mark for a very robust instrumentation technique for interaction measurements. Variation between results is increased only slightly to about 27% when differences in individual users’ data analysis preferences are taken into account.

These findings must be seen in the context of this benchmark study being performed with centrally prepared samples at identical concentrations. For a typical experiment that will be replicated in a different laboratory, combined uncertainties of the actual target and ligand concentration can also approach 20%, i.e. of the order of the major uncertainties found in this benchmark.

As with other measurement techniques, the robustness is strongly dependent on the measurement signal and uncertainty increases significantly for lower signal-to-noise ratios (as can be seen by comparing Tables 1, 3 and 4).

We also provided an estimate for expected relative variation (%) in *K*_D_ for a given S/N of an interaction measured with the Monolith system derived from our analysis of variation across many laboratories. This can be used to readily estimate the effort (number of replicates) to reach a desired relative standard error [Eq. (9)].

For measurements at lower signal-to-noise ratios, estimated confidence intervals will substantially differ depending on the method applied to calculate them, as seen in Figs. 4 and 10. The superior method of calculating confidence intervals by error-surface projection (Bevington and Robinson 1992) instead of using the covariance matrix results will give a more conservative estimate for the precision of the parameters derived from the experiment. This is observed if the question is asked for what proportion of individual measurements does the estimated 68.3% confidence interval contain the mean *K*_D_ of all measurements (i.e. the best estimate of the true *K*_D_) as reported in Table 5.

**Table 5:** Chance for the mean *K*_D_ lying within the 68.3% confidence interval generated by PALMIST or MOAA

	NAG3 (%)	NB Fluo (%)	NB TRIC (%)
PALMIST	77.5	85	92.5
MOAA	62.5	47.5	40

Signal to noise NAG3 > NB Fluo > NB TRIC

The lower the signal-to-noise ratio becomes, the less accurate the confidence interval predictions are by both approaches with the covariance matrix (used by MOAA) progressively further underestimating the likely uncertainty in an individual (triplicate) experiment and the error-surface projection approach (used by PALMIST) further overestimating the uncertainty.

As can be observed in Fig. 10 the *K*_D_ value distributions, and especially the confidence intervals predicted by PALMIST, are clearly asymmetric. Generally, a symmetry for *K*_D_ values cannot be assumed ad hoc both for the confidence interval of the measurement as well as the statistic distribution of measurements since several effects, for instance the design of the experiment (the respective concentrations used in respect to the *K*_D_) will have an influence on the symmetry of both the confidence intervals and statistical distributions. One advantage of PALMISTs way of reporting error confidence intervals is that they directly correspond between *K*_D_ and *ΔG* values*,* while the more commonly reported symmetric uncertainties either in *K*_D_ or *ΔG* can never directly correspond to each other due to their logarithmic relationship. A more detailed insight into proper confidence interval reporting, is discussed in Paketurytė (2021) exemplified for the case of ITC measurements.

This benchmark study revealed a general issue when analyzing TRIC, fluorescence anisotropy (FA) and other datasets that use fluorescence reporters. If the absolute fluorescence is changing upon binding of the ligand it is strongly recommended to directly analyze the fluorescence change [using supplementary equation (ii)] instead of secondary signals like TRIC or FA. Or, to increase the information content available, to analyze the fluorescence intensity change and secondary signals globally using fluorescence weighted corrected models [Eq. (2)]. Unfortunately, this method is not readily available in the current analysis tools which made it difficult to apply to the data from the benchmark study, therefore proper quantification of this effect was not conducted.

Generally, the idea of comparing different experimental methods like SPR, ITC and Monolith instruments with identical test samples is still present in the ARBRE community and will be pursued in the future. However, one key prerequisite for this type of comparison is the availability of a well-defined test system that can be characterized with several techniques under comparable conditions. A model protein–protein interaction not showing fluorescence changes upon binding and a well-defined baseline for saturation, (e.g., new mutants of the lysozyme nanobody) might be the way to proceed.

## Supplementary Information

Below is the link to the electronic supplementary material.

Supplementary file1 (DOCX 199 kb)

Supplementary file2 (PDF 137 kb)

Supplementary file3 (PDF 161 kb)

Supplementary file4 (PDF 60 kb)

## Data Availability

Some of the figures in this report were generated using custom Python scripts. Code is available upon request.
