# Correction to: Reproducibility and accuracy of microscale thermophoresis in the NanoTemper Monolith: a multi laboratory benchmark study

**DOI:** 10.1007/s00249-021-01573-x

**Published:** 2021-10-15

**Authors:** Blanca López-Méndez, Bruno Baron, Chad A. Brautigam, Thomas A. Jowitt, Stefan H. Knauer, Stephan Uebel, Mark A. Williams, Arthur Sedivy, Olga Abian, Celeste Abreu, Malgorzata Adamczyk, Wojciech Bal, Sylvie Berger, Alexander K. Buell, Carlo Carolis, Tina Daviter, Alexander Fish, Maria Garcia-Alai, Christian Guenther, Josef Hamacek, Jitka Holková, Josef Houser, Chris Johnson, Sharon Kelly, Andrew Leech, Caroline Mas, Daumantas Matulis, Stephen H. McLaughlin, Roland Montserret, Rouba Nasreddine, Reine Nehmé, Quyen Nguyen, David Ortega-Alarcón, Kathryn Perez, Katja Pirc, Grzegorz Piszczek, Marjetka Podobnik, Natalia Rodrigo, Jasmina Rokov-Plavec, Susanne Schaefer, Tim Sharpe, June Southall, David Staunton, Pedro Tavares, Ondrej Vanek, Michael Weyand, Di Wu

**Affiliations:** 1 Departamento de Bioquímica y Biología Molecular y Celular-Institute of Biocomputation and Physics of Complex Systems (BIFI), Instituto Aragonés de Ciencias de la Salud (IACS), Instituto de Investigación Sanitaria Aragón (IIS Aragón), Universidad de Zaragoza, C/ Mariano Esquillor S/N, 50018 Zaragoza, Spain; 2 Department of Biochemistry, Faculty of Science, Charles University, Hlavova 8, 128 43 Prague, Czech Republic; 3 Faculty of Chemistry, Chair of Drug and Cosmetics Biotechnology, Warsaw University of Technology, ul. Noakowskiego 3, 00-664 Warsaw, Poland; 4 Institute of Biochemistry and Biophysics, PAS, Pawinskiego 5a, 02-106 Warsaw, Poland; 5 Molecular Biophysics, Institut Pasteur, 25-28 Rue du Dr Roux, 75015 Paris, France; 6 Institut de Recherches Servier, 125, Chemin de Ronde, 78290 Croissy-sur-Seine, France; 7 Departments of Biophysics and Microbiology, UT Southwestern Medical Center, Dallas, TX 75390 USA; 8 Department of Biotechnology and Biomedicine, Technical University of Denmark, Søltofts Plads, Kgs., 2800 Lyngby, Denmark; 9 BioMolecular Screening and Protein Technologies Unit, Centre for Genomic Regulation (CRG), Dr. Aiguader St, 88, 08003 Barcelona, Spain; 10 Department of Biological Sciences, BiophysX Centre, Institute of Structural and Molecular Biology, Birkbeck, University of London, Malet Street, London, WC1E 7HX UK; 11 Present Address: Shared Research Facilities, The Institute of Cancer Research, London, SW7 3RP UK; 12 Department of Biochemistry, Netherlands Cancer Institute, Plesmanlaan 121, 1066CX Amsterdam, Netherlands; 13 EMBL-Hamburg, Notkestrasse 85, 22607 Hamburg, Germany; 14 Center for Molecular Biophysics, UPR 4301 CNRS Orléans, Rue Charles Sadron, 45071 Orléans, France; 15 Glycobiochemistry and Biomolecular Interaction and Crystallization Core Facility, CEITEC MU, Kamenice 5, 625 00 Brno, Czech Republic; 16 MRC Laboratory of Molecular Biology, Francis Crick Avenue, Cambridge Biomedical Campus, Cambridge, CB2 0QH UK; 17 Biomolecular Analysis Core Facility, University of Manchester, Oxford Rd, Manchester, M13 9PL UK; 18 Institute of Molecular, Cell and Systems Biology, University of Glasgow, B4-13 Joseph Black Building, G12 8QQ Glasgow, Scotland, UK; 19 Biochemistry IV-Biopolymers, University of Bayreuth, Universitaetsstr. 30, 95447 Bayreuth, Germany; 20 Department of Biology, Bioscience Technology Facility, University of York, Wentworth Way, York, YO10 5DD UK; 21 Biophysics Platform, Novo Nordisk Foundation Center for Protein Research, University of Copenhagen, 2200 Copenhagen, Denmark; 22 Integrated Structural Biology Grenoble (ISBG), UMS 3518 (CNRS-CEA-UGA-EMBL), 71 avenue des Martyrs, 38042 Grenoble Cedex 9, France; 23 Department of Biothermodynamics and Drug Design, Life Sciences Center, Institute of Biotechnology, Vilnius University, Sauletekio StSaulėtekio 7, 10257 Vilnius, Lithuania; 24 Institut de Biologie et Chimie des protéines, CNRS, Université de Lyon, 7 passage du Vercors, 69367 cedex 07 Lyon, France; 25 Institut de Chimie Organique et Analytique (ICOA), CNRS FR 2708, UMR 7311, Université d’Orléans, Orléans, France; 26 Institute of Biocomputation and Physics of Complex Systems (BIFI), Universidad de Zaragoza, C/ Mariano Esquillor S/N, 50018 Zaragoza, Spain; 27 Biophysics Lab, Protein Expression and Purification Core Facility, EMBL Heidelberg, Meyerhofstraße 1, 69117 Heidelberg, Germany; 28 Department of Molecular Biology and Nanobiotechnology, National Institute of Chemistry, Hajdrihova 19, 1000 Ljubljana, Slovenia; 29 NHLBI Biophysics Core Facility, NHLBI/NIH, 50 South Dr, Bethesda, MD 20892 USA; 30 Division of Biochemistry, Department of Chemistry, Faculty of Science, University of Zagreb, Horvatovac 102a, 10000 Zagreb, Croatia; 31 Department of Biochemistry, University of Bayreuth, Universitätsstr. 30, 95447 Bayreuth, Germany; 32 ProteinTechnology, Vienna Biocenter Core Facilities GmbH, Dr. Bohr-Gasse 3, 1030 Vienna, Austria; 33 Biozentrum, University of Basel, Klingelbergstrasse 50/70, 4056 Basel, Switzerland; 34 Department of Biochemistry, University of Oxford, South Parks Rd, Oxford, OX13 5LA UK; 35 Molecular Biophysics Research Laboratory, Departamento de Química, UCIBIO/Requimte, Faculdade de Ciências e Tecnologia, UNL, Campus Caparica, 2829-516 Costa da Caparica, Portugal; 36 Max Planck Institute of Biochemistry, Am Klopferspitz 18, Martinsried, 82152 Planegg, Germany; 37 Department of Biological Sciences, ISMB BiophysX Centre, Institute of Structural and Molecular Biology, Birkbeck, University of London, London, WC1E 7HX UK

## Correction to: European Biophysics Journal (2021) 50:411–427 10.1007/s00249-021-01532-6

The article “Reproducibility and accuracy of microscale thermophoresis in the NanoTemper Monolith: a multi laboratory benchmark study” written by López-Méndez, B., Baron, B., Brautigam, C. A., Jowitt, T. A., Knauer, S. H., Uebel, S., Williams, M. A., Sedivy, A., Abian, O., Abreu, C., Adamczyk, M., Bal, W., Berger, S., Buell, A. K., Carolis, C., Daviter, T., Fish, A., Garcia-Alai, M., Guenther, C., Hamacek, J., Holková, J., Houser, J., Johnson, C., Kelly, S., Leech, A., Mas, C., Matulis, D., McLaughlin, S. H., Montserret, R., Nasreddine, R., Nehmé, R., Nguyen, Q., Ortega-Alarcón, D., Perez, K., Pirc, K., Piszczek, G., Podobnik, M., Rodrigo, N., Rokov-Plavec, J., Schaefer, S., Sharpe, T., Southall, J., Staunton, D., Tavares, P., Vanek, O., Weyand, M., Wu, D. was originally published Online First without Open Access. After publication in volume 50, issue 3–4, pages 411–427 the author decided to opt for Open Choice and to make the article an Open Access publication. Therefore, the copyright of the article has been changed to © The Author(s) 2021 and the article is forthwith distributed under the terms of the Creative Commons Attribution 4.0 International License, which permits use, sharing, adaptation, distribution and reproduction in any medium or format, as long as you give appropriate credit to the original author(s) and the source, provide a link to the Creative Commons licence, and indicate if changes were made. The images or other third party material in this article are included in the article’s Creative Commons licence, unless indicated otherwise in a credit line to the material. If material is not included in the article’s Creative Commons licence and your intended use is not permitted by statutory regulation or exceeds the permitted use, you will need to obtain permission directly from the copyright holder. To view a copy of this licence, visit http://creativecommons.org/licenses/by/4.0.

The original article has been corrected.

