# Supplementary material for: Reproducibility and accuracy of microscale thermophoresis in the NanoTemper Monolith: a multi laboratory benchmark study
Source: Eur Biophys J. 2021 Apr 21;50(3-4):411–27. doi: 10.1007/s00249-021-01532-6 (PMC8519905; doi:10.1007/s00249-021-01532-6)
Supplement: Supplementary file 1 — Supplementary file1 (DOCX 199 kb) [file 249_2021_1532_MOESM1_ESM.docx]

# Supplementary materials

The law of mass action allows to derive the following relationship between the fraction of bound complex, $X=\frac{c_{AB}}{c_{A, total}}$, for a given *K_D_* with $c_{A, total}$ being the total molar target concentration, $c_{B, total}$ the total ligand concentration and $c_{AB}$ the concentration of the complex.

Supplementary equation (i)

$$X=\frac{c_{B,total}+c_{A,total}+K_{D}-\sqrt{\left( c_{B,total}+c_{A,total}+K_{D} \right)^{2}-4(c_{A,total}+c_{B,total})}}{2c_{A,total}}$$

Assuming the measured *F_norm_* values correspond to a linear superposition of the signals for unbound ($F_{norm}(0))$ and bound $(F_{norm}(1)$) fractions respectively, *F_norm_* values were fitted using supplementary equation (i) and (ii).

Supplementary equation (ii)

$$F_{norm}\left( X \right)=F_{norm}(0)+X\cdot\left( F_{norm}(1)-F_{norm}(0) \right)$$

| dye | buffer | *K_D_* [µM] | *σ* [µM] | Fluorescence change upon binding |
| --- | --- | --- | --- | --- |
| No dye (LabelFree) | sodium acetate+ ^a)^ | 4.1 | 1.1 | NO |
| Blue-NHS (Dylight 488) | sodium acetate+ ^a)^ | 10.0 | 1.8 | YES |
| Blue-NHS  (Dylight 488) | PBS+ | 17.8 | 2.2 | YES |
| NT-RED-NHS | PBS+ | 97.6 | 18.4 | NO |
| NT-RED-NHS 2^nd^ gen. | PBS+ | 25.5-43.2 ^b)^ | ~5 | NO |
| NT-RED-NHS 2^nd^ gen. | TRIS+ | 47.9 | 2.7 | NO |

Supplementary table 1: list of labeling strategies and buffers tested for the lysozyme–NAG3 interaction prior to the 40-instrument benchmark. ^a)^ Sodium acetate + buffer: 100mM sodium acetate pH5.0, 0.1% Tween-20. ^b)^ Different labeling replicates with different labeling efficiencies.

b)

a)

Supplementary figure 1: a) *F_norm_* values derived in MOAA for 25 nM RED-NHS 2^nd^ generation dye of each individual instrument for 20, 40 and 60% MST power respectively. b) Values from a) plotted as function of instrument manufacturing year.

Supplementary figure 2: *K_D_* versus *F_norm_* values of the unbound state ($F_{norm}(0)$) derived in MOAA for Lysozyme–NAG3 interaction.


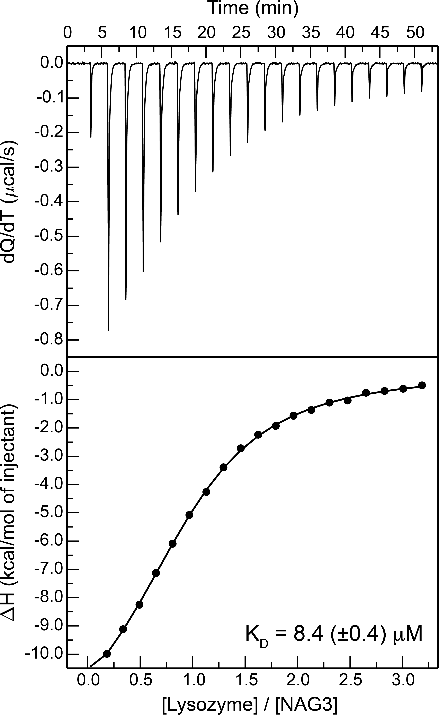


Supplementary figure 3: Exemplary ITC measurement for the lysozyme–NAG3 interaction in 20 mM Tris pH 7.8, 150 mM NaCl, 0.005% Tween-20 at 25°C. 29 µM Lysozyme in the cell and 450 µM NAG3 in the syringe measured with a MicroCal Auto-iTC200, analyzed with the MicroCal PEAQ-ITC analysis software.

Supplementary figure 4: Distribution of extracted *K_D_* values for the Lysozyme–NAG3 interaction regarding the S/N ratio of the replicates for all measurements. The horizontal line corresponds to the average *K_D_* of 54.8µM while the dashed lines correspond to the S/N dependent relative standard deviation of triplicates as estimated in equation (8).


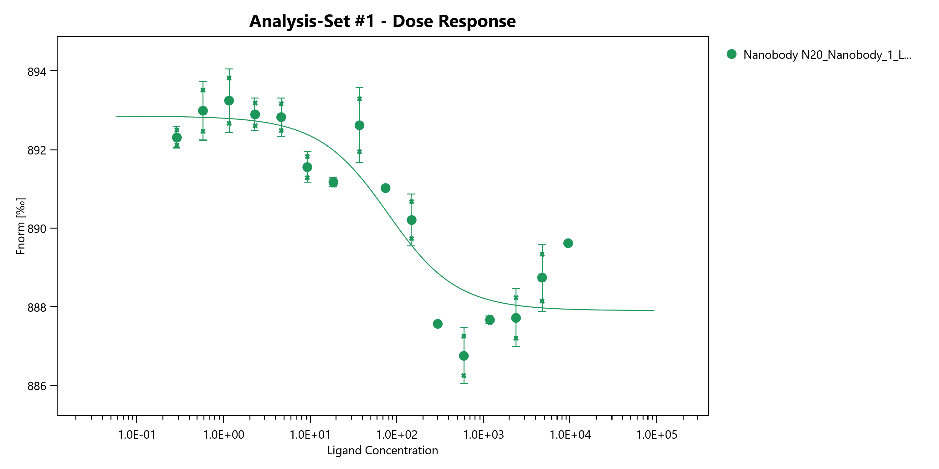


Supplementary figure 5: Exemplary TRIC binding curve for the lysozyme–NB interaction in PBS+, data from instrument N20. For high ligand concentration non-ideal behavior can be seen with the upper baseline deviation from the expected 1:1 binding model. Graph exported from MOAA.


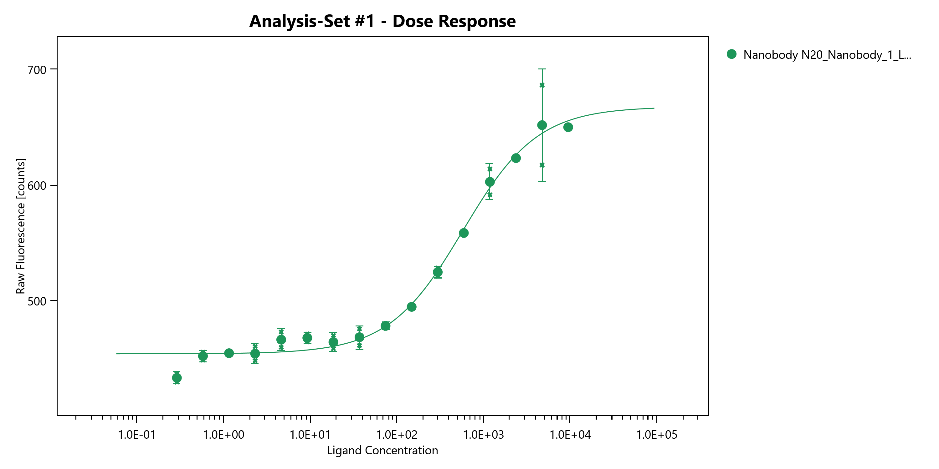


Supplementary figure 6: Exemplary binding curve from the fluorescence analysis for the lysozyme–NB interaction in PBS+, data from instrument N20. Curve nicely resembles a 1:1 binding model. Graph exported from MOAA.
