# Supplementary material for: Reproducibility and accuracy of microscale thermophoresis in the NanoTemper Monolith: a multi laboratory benchmark study
Source: Eur Biophys J. 2021 Apr 21;50(3-4):411–27. doi: 10.1007/s00249-021-01532-6 (PMC8519905; doi:10.1007/s00249-021-01532-6)
Supplement: Supplementary file 2 — Supplementary file2 (PDF 137 kb) [file 249_2021_1532_MOESM2_ESM.pdf]

MST benchmark standard operating procedure (SOP) v 1.1

Content:

For each instrument to benchmark, one envelope containing the following samples in 2 individual plastic bags (one for each system) are provided.

Label	Plastic bag	Sample	Concentration
Lysozyme-RED_PBS+	1	Lysozyme from chicken egg white red labelled (RED-NHS 2nd Generation dye) in PBS+	50 nM
Nanobody	1	Nanobody against lysozyme	19 µM
PBS+	1	PBS, 0.005% Tween-20	-
Lysozyme-RED_Tris+	2	Lysozyme from chicken egg white red labelled (RED-NHS 2nd Generation dye) in Tris+	50 nM
NAG3	2	N,N',N''-Triacetylchitotriose in Tris+	2 mM
Tris+	2	20 mM Tris pH 7.8, 150 mM NaCl, 0.005% Tween-20	-
RED-NHS-II dye	1	RED-NHS 2nd Generation dye (NanoTemper Technologies GmbH) in PBS+	25 nM

Samples for interaction measurement (Lysozyme, NAG3/Nanobody, respective buffer) come in triplicates. The lysozyme NAG3 interaction will be measured in Tris+ buffer while the lysozyme nanobody interaction will be measured in PBS+ buffer. Please make sure not to confuse the Lysozyme-RED samples with each other since they are already in the respective buffers!

The RED dye sample for instrument calibration will be measured in 5 capillaries. The same capillaries will be measured at different MST power.

Storage:

Store all samples at 4°C for no longer than 3 weeks.

Naming convention:

For each instrument to benchmark you will receive a code from Stefan Knaur containing a Letter and two numbers. This code will be an anonymous and unique identifier. NT.115 instruments will have an "N" as letter while pico instruments will have a "P" as letter. Since some instruments will be operated

by the NT Control software and some instruments by the MO.Control software both types are covered in this SOP. Naming of the file ("Project" in NT Control or "Session" in MO.Control) as well as each single experiment and experimental parameters should follow the outline below. Make sure that there is no name or abbreviation in your data that hints at your identity. Please use only a single file in which all the experiments are saved in the respective order, so that automated analysis of the files will be easier.

Order of experiments:

1. Lysozyme Nanobody interaction replicate 1
2. Lysozyme Nanobody interaction replicate 2
3. Lysozyme Nanobody interaction replicate 3
4. Lysozyme NAG3 interaction replicate 1
5. Lysozyme NAG3 interaction replicate 2
6. Lysozyme NAG3 interaction replicate 3
7. Instrument calibration low MST power
8. Instrument calibration medium MST power
9. Instrument calibration high MST power

How to fill in parameters (NT Control / MO.Control):

Project name / Session name: participant code (= *CODE*), e.g. N01

(in this case either a N01.ntp or N01.moc file is generated)

Nanobody interaction:

Experiment name: (*CODE*_Nanobody_*#replicate*), e.g. N01_Nanobody_2

Temperature: 37°C

Target: Lysozyme (nothing to fill in in NT Control)

Name/Ligand: Nanobody

Concentration Fluor. Mol./Target concentration: 25 nM

Ligand concentration series: 1:1 series starting at 9500 nM

Buffer: PBS+ (nothing to fill in in NT Control)

NAG3 interaction:

Experiment name: (*CODE*_NAG3_*#replicate*), e.g. N01_NAG3_2

Temperature: 25°C

Target: Lysozyme (nothing to fill in in NT Control)

Ligand: NAG3

Target concentration: 25 nM

Ligand concentration series: 1:1 series starting at 1 000 000 nM

Buffer: TRIS+ (nothing to fill in in NT Control)

Instrument calibration:

Experiment name: (*CODE*_calibration for NT Control or *CODE*_calibration_low, *CODE*_calibration_medium and *CODE*_calibration_high for MO.Control), e.g. N01_calibration_medium

Temperature: 25°C

Target: dye (nothing to fill out in NT Control)

Ligand: none

Target concentration: 25 nM

Ligand concentration series: 1:1 series starting at 1.000 nM

Buffer: PBS+ (nothing to fill in in NT Control)

Preparation prior to measurements:

All samples:

Prior to usage, mix each sample by pipetting up and down several times. Spin the sample down using a benchtop centrifuge for 10 min at max speed (~10.000 x *g*). Transfer the supernatant to a fresh tube.

Sample preparation (for the interaction measurements):

Preparation of ligand dilution series (for both interaction measurements, each in triplicates using the respective buffer):

Only use one pipette and don’t change the volume of this pipette throughout the whole preparation!

i. Set the pipetting volume to 10µl (and clamp it if possible with your pipette).
ii. Prepare 16 PCR tubes and fill tubes 2 to 16 with 10µl of the respective buffer by reverse pipetting, leaving tube 1 empty. Instead of 16 single PCR tubes two PCR strips each with 8 tubes can be used. One pipette tip should be used to fill all 15 tubes.

> Reverse Pipetting:
>
> 1. Push down the pipette piston to the second stop.
> 2. Put the tip into the liquid and release the piston to fill the pipette (more than 10µl are now in the tip).
> 3. Put the pipette tip to the respective tube, touch the bottom and push down the piston to the first stop of the pipette (just 10µl are released).

iii. Use a fresh tip and put 10µl of the ligand from the stock to tube 1 (using “regular” pipetting), no prior dilution of the ligand is required.
iv. Use the same tip and put 10µl of the ligand from the stock to tube 2 and mix it with at least four up/down strokes of the pipette piston.
v. Take a new tip and retrieve 10µl from tube 2, put it to tube 3 and mix it with at least four up/down strokes of the pipette piston.
vi. Repeat step v. until tube 16 is filled with ligand.
vii. Remove 10µl of tube 16 so it only contains 10µl as every other tube.

Prepare solutions for measurement:

Use the identical pipette that was not changed in volume as was used for the preparation of the dilution series!

i. Add 10µl of labelled lysozyme sample (the target) from the stock solution to tube 16 of the dilution series (lowest concentration of ligand) and mix it with at least four up/down strokes of the pipette piston. Make sure to use the labelled lysozyme in Tris+ for NAG3, and the labelled lysozyme in PBS+ for the nanobody!
ii. Repeat i. for tubes 15-1 (from lower to higher concentration of ligand) until all (16) tubes contain 10µl of labelled protein with the same tip (no need to change tips if going from low to high concentration).

Filling of capillaries:

Only touch the capillaries on the end that is not going to be in contact with the measured solvents!

i. Single tubes:
    a. Take the tube in one hand and the capillary into the other hand.
    b. Tilt the tube to about 45° and insert the capillary into the measurement solvent until it is filled well above the center. (If this is not working properly tilt the capillary/tube even more, if this still doesn’t help use another capillary)
ii. PCR strips:
    a. Put a capillary in every tube of the PCR strip.
    b. Gently take the PCR strip in one hand and tilt it until all capillaries are filled well above the center (the other hand can help by moving the capillaries a little, only touching them at the end).
    c. Remove the capillaries one by one with one hand while holding the PCR strip in the other.
iii. Make sure there is no droplet on the outside of the capillary (if there is, wipe it off by touching the edge of the PCR tube with the capillary).
iv. Place the capillary in the capillary tray and fix it if possible by sliding the magnetic holder over it (If your instrument does not have a capillary holder you have to place the capillaries very carefully).
v. After the whole capillary tray is filled, fix the capillaries either using the magnetic holders or by closing the holder lid.
vi. Put the capillary tray into the instrument.

Make sure the central part of the capillaries is filled with liquid, does not contain any air bubbles and no liquid is “crawling” on the outside of the capillary!

Starting the measurement:

Place the capillary holder in the instrument, close it and prepare a measurement in the control software. Use the protocol below, depending on which control software is used in your lab (NT Control or MO.Control).

NT Control (v2.0.2.29):

i. Create a new Project with the name listed above in the naming conventions.
ii. Enable Manual Temperature control and set temperature to 37°C for the nanobody interaction or 25°C for the NAG3 interaction (make sure the On/Off button in the software is orange and the target temperature is displayed on the instrument display).
iii. Create a single run in the table of runs using the following parameters:
    a. LED Color: RED (for Monolith NT.115)
    b. From Cap No: 1, To Cap No: 16
    c. LED Power: 60% for Monolith NT.115, 2% for Monolith Pico
    d. MST Power: 40%
    e. Fluo. Before: 5s, MST on 30s, Fluo. After 5s
    f. Delay: 25s
iv. Fill in the Experiment's Name using the respective names listed above in the naming conventions.
v. Fill in the concentration of Fluor. Mol. using the numbers listed above.
vi. Fill in the concentration at the table of Capillaries using the highest concentration stated above (i.e., 9500 nM for the nabody and 1000000 nM for the NAG3) and fill the rest of the column using the 1:1 dilution option.
vii. Fill in the Names at the table of Capillaries using the names listed above.
viii. Start a Cap Scan and check the measured fluorescence. Ideally it should be between 500 and 1500 counts for the Monolith NT.115 and between 5000 and 15000 counts for the Monolith Pico.

If the counts are lower than in the range stated above, check if the correct LED Settings are applied. If this is fine increase the LED power until you reach a reasonable signal. If this is not possible, please contact the benchmark coordinator.

If the counts are above the range stated above, reduce the LED power until you reach a reasonable signal. Add "_LED_adjust" to the experiment name if you had to change the LED power.

ix. Wait until the temperature on the instrument display reached the set temperature (or wait at least 5 minutes when using fresh capillaries).
x. Start the measurement.

Repeat the sample preparation and measurement **two more times** for the same sample to get **triplicates**, adjusting the experiment names accordingly.

Redo the **triplicate measurements** according to the protocol above for the **second interaction system**.

MO.Control (v1.6):

i. Create a New Session with the name listed above in the naming conventions.
ii. Choose the Nano – RED or Pico – RED respectively.
iii. Create a New Experiment in Expert mode.
iv. Change the name of the experiment (left hand side using the pen) to the names listed above.
v. Set the Temperature Control and to 37°C for the nanobody interaction or 25°C for the NAG3 interaction.
vi. Fill in the following parameters:
    a. From Capillary: 1, To Capillary: 16
    b. Excitation Power: 60% for Monolith NT.115, 2% for Monolith Pico
    c. MST-Power: Medium
    d. Before MST: 5s, MST-On Time: 30s, After MST: 5s
vii. Add a Dilution Series (1:1) using the respective Ligand and Target names as well as the concentrations stated above in the naming convention and the respective buffer names.
viii. Start the measurement.

If the LED counts are out of range, the software will tell you. If the counts are lower than in the range stated above, check if the correct LED Settings are applied. If this is fine increase the LED power until you reach a reasonable signal. If this is not possible, please contact the benchmark coordinator.

If the counts are above the range stated above, reduce the LED power until you reach a reasonable signal.

If you had to adjust the LED power, start a new measurements and add "_LED_adjust" to the experiment name.

Repeat the sample preparation and measurement **two more times** for the same sample to get **triplicates**, adjusting the experiment names accordingly.

Redo the **triplicate measurements** according to the protocol above for the **second interaction sample**.

Sample preparation (instrument calibration sample):

To calibrate the instrument (both absolute fluorescence counts and MST power), pure dye is measured. Mix the calibration sample by pipetting up and down several times. Spin the sample down using a benchtop centrifuge for 10 min at max speed (~10.000 x *g*). Transfer the supernatant to a new tube.

Fill 5 capillaries with the same sample, one by one, according to the protocol above (filling of capillaries).

Starting the measurement (instrument calibration sample):

Place the capillary holder in the instrument, close it and prepare a measurement in the control software.

NT Control (v2.0.2.29):

i. Load the Project used for the interaction measurements or just continue if the file is already open.
ii. Enable Manual Temperature control and set temperature to 25°C (make sure the On/Off button in the software is orange and the target temperature is displayed on the instrument display).
iii. Create three runs in the table of runs using the following parameters:
    a. LED Color: RED (for Monolith NT.115)
    b. From Cap No: 1, To Cap No: 5
    c. LED Power: 60% for Monolith NT.115, 2% for Monolith Pico
    d. MST Power: 20%, 40% and 60% for each run respectively
    e. Fluo. Before: 5s, MST on 30s, Fluo. After 5s
    f. Delay: 25s
iv. Fill in the Experiment's Name using the calibration name listed above in the naming conventions.
v. Fill in the Concentration at the table of Capillaries using 1000 for the highest concentration and fill the rest of the column using the 1:1 dilution option.
vi. Fill in the Names at the table of Capillaries using "none" as the name for the ligand.
vii. Start a Cap Scan and check the measured fluorescence. If the fluorescence is not between 500 and 1500 counts for the Monolith NT.115 and between 5000 and 15000 counts for the Monolith Pico, just do a single run (at 20%MST power) instead.
viii. Wait until the temperature on the instrument display reached the set temperature (or wait at least 5 minutes when using fresh capillaries).
ix. Start the measurement.

If the fluorescent counts were not in the range stated above, do another experiment with adjusted LED setting. If the counts were too low, increase the LED power until you reach a reasonable signal. If this is not possible, please contact the benchmark coordinator.

If the counts were above the range stated above, reduce the LED power until you reach a reasonable signal.

For a second (adjusted LED power) run, create the three runs mentioned above and add "_LED_adjust" to your experiment's name and start the adjusted measurement.

MO.Control (v1.6):

i. Browse the previous Session used for the interaction measurements or continue if the file is already open.
ii. Choose the Nano – RED or Pico – RED respectively.
iii. Create a New Experiment in Expert mode.
iv. Change the name of the experiment (left hand side using the pen) to the calibration names listed above in the naming conventions.
v. Set the Temperature Control to 25°C.
vi. Click 'Add a Dilution Series' (1:1) using none as Ligand and dye as Target names,
vii. 25 nM as Target concentration, 1000nM as highest Ligand concentration and PBS+ as buffer name.
viii. Fill in the rest using following parameters:
   a. From Capillary: 1, To Capillary: 5
   b. Excitation Power: 60% for Monolith NT.115, 2% for Monolith Pico
   c. MST-Power: Low (Medium/High – three independent experiments!)
   d. Before MST: 5s, MST-On Time: 30s, After MST: 5s
ix. Start the measurement.

If the LED counts are out of range, the software will tell you. If the counts are lower than in the range stated above, check if the correct LED Settings are applied. If this is fine increase the LED power until you reach a reasonable signal. If this is not possible, please contact the benchmark coordinator.

If the counts are above the range stated above, reduce the LED power until you reach a reasonable signal. Add "_LED_adjust" to the experiment name if you had to change the LED power.

i. Leave the capillaries in the instrument, create and measure two more experiments with Medium and with High MST power, changing the experiment names according to the instruction sheet.
