# Supplementary material for: Reproducibility and accuracy of microscale thermophoresis in the NanoTemper Monolith: a multi laboratory benchmark study
Source: Eur Biophys J. 2021 Apr 21;50(3-4):411–27. doi: 10.1007/s00249-021-01532-6 (PMC8519905; doi:10.1007/s00249-021-01532-6)
Supplement: Supplementary file 3 — Supplementary file3 (PDF 161 kb) [file 249_2021_1532_MOESM3_ESM.pdf]

MST benchmark information

Please fill in this information sheet as soon as you finished the experiments/data analysis. Make sure no personal information is given within this file. Save this file using the unique code for the instrument you were give as name, e.g. N05.docx.

Metadata about the experiment

Unique CODE: .

Type of instrument (NT.115/pico), if NT.115 which filter sets are available: .

Instrument, date of purchase (Year): .

Instrument serial number (on the back of the instrument, e.g. S/N: 201403-BR-N002): .

Format of the tray (magnetic strips/complete cover): .

Date sample arrived to your lab/put to 4°C: .

Software used for measurements (NTControl/MO.Control) including version number (e.g. written on the left side of the SW main window on top of the menu bar):

.

Date(s) of samples measurements:

Nanobody_1: .

Nanobody_2: .

Nanobody_3: .

NAG3_1: .

NAG3_2: .

NAG3_3: .

Dye: .

Temperature of the room where the instrument is in: .

Adjusted LED power (if not the LED power mentioned in the SOP was used):

For lysozyme – nanobody interaction: .

For lysozyme – NAG3 interaction: .

For dye /calibration measurements: .

Measurement analysis

Please analyze the two interactions according to how you usually analyze MST measurements. Please fill in the questions below about the way you analyze the data and the results for both interactions. When plots or numbers are asked you can just copy-paste a snapshot of the results from your analysis tool.

Software used for data analysis (NTAnalysis/MO.Analysis/PALMIST/other) including version number:

General comments (did you need to redo an experiment? issues during the experiment/temperature control? …):

Lysozyme – nanobody interaction

Plot of the raw MST curves showing the regions used for data analysis (hot/cold region):

Plot of binding curve showing the fit:

How did you analyze the data / how did you choose the region to analyze? (software chooses automatically, manually chosen region because …) Please give a short paragraph about how you typically analyze MST data:

Results (please report numbers retrieved from the software you used, if the software does not report those numbers please give an estimate):

Hot region (from to): .

Cold region (from to): .

K_D / accuracies: .

Amplitude: .

Noise: .

Lysozyme – NAG3 interaction

Plot of the raw MST curves showing the regions used for data analysis (hot/cold region):

Plot of binding curve showing the fit:

How did you analyze the data / how did you choose the region to analyze? (software chooses automatically, manually chosen region because …) Please give a short paragraph about how you typically analyze MST data:

Results (please report numbers retrieved from the software you used, if the software does not report those numbers please give an estimate):

Hot region (from to): .

Cold region (from to): .

K_D / accuracies: .

Amplitude: .

Noise: .
