# Supplementary material for: Reproducibility and accuracy of microscale thermophoresis in the NanoTemper Monolith: a multi laboratory benchmark study
Source: Eur Biophys J. 2021 Apr 21;50(3-4):411–27. doi: 10.1007/s00249-021-01532-6 (PMC8519905; doi:10.1007/s00249-021-01532-6)
Supplement: Supplementary file 4 — Supplementary file4 (PDF 60 kb) [file 249_2021_1532_MOESM4_ESM.pdf]

ARBRE-MOBIEU benchmark of Monolith NT.115 instruments

Fnorm spread

Based on the findings of the ARBRE benchmark we have developed a novel QC procedure, including a calibration sample that will yield a higher instrument to instrument reproducibility. Please note that the variability between different instruments is unrelated to the variability within one instrument. It is also noteworthy that the distribution of absolute Fnorm values does neither correlate with the obtained dissociation constants, nor does it significantly correlate with the amplitude of the measured interaction. Even for a µM affinity the dissociation constants obtained were in excellent agreement from instrument to instrument, thus a disturbance of the thermodynamic equilibrium by small temperature differences can be excluded.

Y-offset correction

When merging MST data in MO.Affinity Analysis software, a Y-offset correction of the given data sets is performed. The correction allows to compensate for slight offsets between different measurements.

When two or more runs are merged, MO.Affinity Analysis computes the average response value for each ligand concentration, resulting in an average dose-response curve. For each measured data point, the vertical offset is computed with respect to the corresponding data point of the average dose-response curve. Next, the average offset for each run is subtracted from the measured data point, shifting the whole run vertically by the same value. Response values of the merged dose-response curve are computed as the average of the shifted dose-response curve at each ligand concentration. Error bars for each data point on the merged dose-response curve are calculated as the standard deviation of the shifted responses as each ligand concentration.

The Y-offset correction does not affect the calculated K_d-value nor the signal amplitude.
